# Characterization of a novel GH30 non-specific endoxylanase *Ac*Xyn30B from *Acetivibrio clariflavus*

**DOI:** 10.1007/s00253-024-13155-w

**Published:** 2024-04-29

**Authors:** Katarína Šuchová, Walid Fathallah, Vladimír Puchart

**Affiliations:** 1 Institute of Chemistry, Slovak Academy of Sciences, Dúbravská cesta 9, 845 38 Bratislava, Slovakia; 2 Faculty of Science, Beni-Suef University, Beni-Suef, 625 11 Egypt

**Keywords:** Non-specific xylanase, *Acetivibrio clariflavus*, Glycoside hydrolase family 30, New subfamily GH30_12, Glucuronoxylan, Arabinoxylan

## Abstract

**Abstract:**

The xylanolytic enzymes Clocl_1795 and Clocl_2746 from glycoside hydrolase (GH) family 30 are highly abundant in the hemicellulolytic system of *Acetivibrio clariflavus* (*Hungateiclostridium*, *Clostridium clariflavum*). Clocl_1795 has been shown to be a xylobiohydrolase *Ac*Xbh30A releasing xylobiose from the non-reducing end of xylan and xylooligosaccharides. In this work, biochemical characterization of Clocl_2746 is presented. The protein, designated *Ac*Xyn30B, shows low sequence similarity to other GH30 members and phylogenetic analysis revealed that *Ac*Xyn30B and related proteins form a separate clade that is proposed to be a new subfamily GH30_12. *Ac*Xyn30B exhibits similar specific activity on glucuronoxylan, arabinoxylan, and aryl glycosides of linear xylooligosaccharides suggesting that it is a non-specific xylanase. From polymeric substrates, it releases the fragments of degrees of polymerization (DP) 2-6. Hydrolysis of different xylooligosaccharides indicates that *Ac*Xyn30B requires at least four occupied catalytic subsites for effective cleavage. The ability of the enzyme to hydrolyze a wide range of substrates is interesting for biotechnological applications. In addition to subfamilies GH30_7, GH30_8, and GH30_10, the newly proposed subfamily GH30_12 further widens the spectrum of GH30 subfamilies containing xylanolytic enzymes.

**Key points:**

- *Bacterial GH30 endoxylanase from A. clariflavus (AcXyn30B) has been characterized*
- *AcXyn30B is non-specific xylanase hydrolyzing various xylans and xylooligosaccharides*
- *Phylogenetic analysis placed AcXyn30B in a new GH30_12 subfamily*

**Supplementary Information:**

The online version contains supplementary material available at 10.1007/s00253-024-13155-w.

## Introduction

Enzymes active on carbohydrates are grouped in CAZy database (https://www.cazy.org) where they are classified into families according to their primary, secondary, and tertiary structure similarity (Drula et al. 2022). Glycoside hydrolase family 30 (GH30) is currently divided into 10 subfamilies and contains diverse enzymatic activities including β-glucocerebrosidases (GH30_1), β-glucosidases (GH30_1, 6), β-1,6-glucanases (GH30_3), β-xylosidases (GH30_2), β-1,4-endoxylanases (GH30_7, 8), reducing end xylose releasing β-1,4-exoxylanases (GH30_7), β-1,4-xylobiohydrolases (GH30_7, 10), β-1,6-galactanases (GH30_5), β-glucuronidases (GH30_9), and β-fucosidases (GH30_4). A new subfamily GH30_11 has been proposed just recently and contains β-1,6-galactobiohydrolases (Li et al. 2022).

GH30 enzymes active on xylan have so far been found in the subfamilies GH30_7, GH30_8, and GH30_10 (Puchart et al. 2021). While bacterial GH30_8 members are mostly specific glucuronoxylanases (EC 3.2.1.136) requiring MeGlcA substitution of the xylan chain for their activity (St. John et al. 2006; Vršanská et al. 2007), fungal GH30_7 members show broader substrate specificity (Šuchová et al. 2021b). In addition to specific glucuronoxylanases (Biely et al. 2014), the GH30_7 subfamily contains non-specific endo-β-1,4-xylanases (EC 3.2.1.8) (Nakamichi et al. 2019c; Šuchová et al. 2021c), reducing end xylose releasing xylanases (Rex-es, EC 3.2.1.156) (Tenkanen et al. 2013; Nakamichi et al. 2019a), xylobiohydrolases (acting at the non-reducing end) (Šuchová et al. 2020) and bifunctional glucuronoxylanases/xylobiohydrolases (Nakamichi et al. 2019b; Katsimpouras et al. 2019). The GH30_10 subfamily was established just recently, and it contains bacterial xylobiohydrolases (Šuchová et al. 2021a; Crooks et al. 2021).

*Acetivibrio clariflavus* (basonym: *Clostridium clariflavum, Hungateiclostridium clariflavum*) is a Gram-positive, thermophilic, cellulolytic cellulosome-forming bacterium, isolated from an anaerobic sewage sludge (Shiratori et al. 2009). Analysis of the *A. clariflavus* cellulosome has shown that the GH30 enzymes (Clocl_1795, Clocl_2746) were highly abundant in all cellulosome fractions examined (Artzi et al. 2015). Both enzymes exhibited xylanolytic activity. Recently it was found that Clocl_1795 is a xylobiohydrolase *Ac*Xbh30A releasing xylobiose from the non-reducing end of xylan and xylooligosaccharides (Šuchová et al. 2021a; Crooks et al. 2021). The enzyme was crystallized, its 3-D structure was solved and the interactions of xylobiose with the enzyme active site were identified (St John et al. 2022). The *Ac*Xbh30A became a founding member of the GH30_10 subfamily. However, the second GH30 xylanolytic enzyme from *A. clariflavus* (Clocl_2746, AEV69300.1) has not been studied yet. Here we report its characterization, and we show that it is a non-specific endo-β-1,4-xylanase *Ac*Xyn30B. Based on phylogenetic analysis that distinguishes *Ac*Xyn30B and related proteins from other GH30 members, we propose to establish a new subfamily GH30_12.

## Materials and methods

### Substrates, standards, and enzymes

Beechwood 4-*O*-methylglucuronoxylan (GX) was prepared as described earlier (Ebringerová et al. 1967). Rhodymenan (Rho), an algal linear β-1,3-β-1,4-xylan from *Palmaria palmata*, was a gift from Prof. M. Claeyssens (University of Ghent, Ghent, Belgium). Wheat arabinoxylan (AraX, Ara:Xyl 38:62, medium viscosity), 4-nitrophenyl glycosides of xylobiose and xylotriose, linear β-1,4-xylooligosaccharides (Xyl_2_-Xyl_6_), arabinoxylooligosaccharides A^3^X (α-l-Ara*f*-1,3-β-d-Xyl*p*-1,4-β-d-Xyl*p*), A^2^XX (α-l-Ara*f*-1,2-β-d-Xyl*p*-1,4-β-d-Xyl*p*-1,4-β-d-Xyl*p*), A^2+3^XX (α-l-Ara*f*-1,3-[α-l-Ara*f*-1,2]-β-d-Xyl*p*-1,4-β-d-Xyl*p*-1,4-β-d-Xyl*p*), XA^3^XX (β-d-Xyl*p*-1,4-[α-l-Ara*f*-1,3]-β-d-Xyl*p*-1,4-β-d-Xyl*p*-1,4-β-d-Xyl*p*), a mixture of XA^3^XX and XA^2^XX (β-d-Xyl*p*-1,4-[α-l-Ara*f*-1,2]-β-d-Xyl*p*-1,4-β-d-Xyl*p*-1,4-β-d-Xyl*p*), XA^2+3^XX (β-d-Xyl*p*-1,4-[α-l-Ara*f*-1,3]-[α-l-Ara*f*-1,2]-β-d-Xyl*p*-1,4-β-d-Xyl*p*-1,4-β-d-Xyl*p*) and GH67 α-glucuronidase from *Geobacillus stearothermophilus* (E-AGUBS) were purchased from Megazyme International (Wicklow, Ireland). Xylose was from Serva (Heidelberg, Germany). MeGlcA^3^Xyl_3_ and MeGlcA^3^Xyl_4_ were prepared from beechwood GX as described previously (Biely et al. 1997). GH3 β-xylosidase was a recombinant *Aspergillus niger* enzyme expressed in *Saccharomyces cerevisiae* (Biely et al. 2000). *A. clariflavus Ac*Xyn30B (product number: CZ0917) was purchased from NZYTech (Lisboa, Portugal).

### Amino acid sequence and phylogenetic analysis

Proteins homologous to *Ac*Xyn30B (GenBank: AEV69300.1, Uniprot: G8M2Z1) were searched using the BlastP (https://blast.ncbi.nlm.nih.gov/Blast.cgi). Characterized GH30 enzymes were collected from CAZy database (http://www.cazy.org/GH30.html). For amino acid comparison and phylogenetic analysis, only the sequences of catalytic domains were used. Selected representatives from each GH30 subfamily were aligned using Clustal Omega server (Madeira et al. 2019) and Supplemental Fig. S1 was prepared using ESPript server (Robert and Gouet 2014). For phylogenetic analysis, 47 amino acid sequences of characterized GH30 members and 14 amino acid sequences of proteins most similar to *Ac*Xyn30B were firstly aligned in Clustal Omega, and the alignment was then analyzed in MEGAX using the Maximum Likelihood method and JTT matrix-based model (Jones et al. 1992; Kumar et al. 2018). Phylogenetic tree branch support values were obtained with 500 cycles of bootstrap analysis (Felsenstein 1985).

### Hydrolysis of polysaccharides and oligosaccharides

Specific activity on polysaccharides was determined on the basis of released reducing sugars quantified by Somogyi-Nelson procedure (Paleg 1959). 10 mg.ml^−1^ solutions of GX, Rho, and AraX in 50 mM sodium phosphate buffer, pH 6, were mixed with *Ac*Xyn30B (final concentration 30.9 nM) and incubated at 40 °C. At time intervals 200 μl aliquots were taken for analysis. 2 mM solutions of 4-nitrophenyl glycosides (NP-Xyl_3_, NP-Xyl_2_) in 50 mM sodium phosphate buffer, pH 6, were incubated with 22.7 nM *Ac*Xyn30B at 40 °C and release of 4-nitrophenol was recorded for 1 h in 5 min intervals by measuring the absorbance at 410 nm. One unit of activity is defined as the amount of the enzyme liberating in 1 min 1 μmol of 4-nitrophenol (from the chromogenic NP-glycosides) or 1 μmol of reducing sugars expressed as an equivalent of xylose.

For thin layer chromatography (TLC) analysis, 10 mg.ml^−1^ polysaccharide solutions (GX, Rho, AraX) were incubated with 0.27 μM *Ac*Xyn30B at 40 °C. Aliquots of 5 μl were spotted on silica gel-coated aluminum sheets (Merck, Darmstadt, Germany) after 10 min, 1 h, 5 h, and 24 h of hydrolysis. The reaction was terminated after 24 h by heating at 100 °C for 5 min. Subsequent treatment with β-xylosidase (1 U.ml^−1^) was done overnight at 40 °C after adjusting pH of the hydrolysates to 4.0 with 4 M acetic acid (due to a lower pH optimum of the β-xylosidase). The treatment with GH67 α-glucuronidase (10 U/ml) was done overnight at 40 °C and pH 6. Hydrolysis of oligosaccharides was done with 2 mM solutions of xylooligosaccharides (XOs) (Xyl_2_ – Xyl_6_), arabinoxylooligosaccharides (A^3^X, A^2^XX, A^2+3^XX, XA^3^XX, XA^2+3^XX and the mixture of XA^3^XX+XA^2^XX) or acidic XOs (MeGlcA^3^Xyl_3_ and MeGlcA^3^Xyl_4_) in 50 mM phosphate buffer, pH 6, with 0.14 μM *Ac*Xyn30B. 2 μl of the mixtures were spotted onto the TLC plate after 10 min, 1 h, 5 h and 24 h of hydrolysis at 40 °C. TLC plates were developed twice for linear XOs Xyl_2_ – Xyl_6_ and once for the acidic XOs in the solvent system ethyl acetate/acetic acid/2-propanol/formic acid/water 25:10:5:1:15 (v/v). TLC plates with arabino-XOs were developed once in the solvent system *n*-butanol/ethanol/water 10:8:5 (v/v). In all cases, the sugars were visualized using orcinol reagent (0.5% orcinol in 5% sulfuric acid in ethanol) and a heating at 105 °C.

### Determination of kinetic constants

Kinetic parameters for GX and AraX hydrolysis were determined at 40 °C in 50 mM sodium phosphate buffer, pH 6. For GX (0.5–20 mg.ml^−1^) and AraX (1–20 mg.ml^−1^), the amount of released reducing sugars was determined at several time points by Somogyi-Nelson procedure (Paleg 1959). For NP-Xyl_2_ and NP-Xyl_3_ (both 0.05–1 mM), the release of 4-nitrophenol was followed for 1 h, by measuring the changes in absorbance at 410 nm. Kinetic constants were calculated by a non-linear regression using Origin 6.0 program (OriginLab Corp., Northampton, MA, USA).

### Matrix-assisted laser desorption ionization–time of flight mass spectrometry (MALDI–TOF MS)

The hydrolysates were decationized by Dowex 50 (H+ form) and 1 μl was mixed with 1 μl of the matrix (1% solution of 2,5-dihydroxybenzoic acid in 30% acetonitrile) directly on the MS target plate. After air-drying, the samples were analyzed by UltrafleXtreme MALDI TOF/TOF mass spectrometer (Bruker Daltonics, Bremen, Germany) operating in reflectron positive mode.

## Results

### Amino acid sequence comparison

**Fig. 1:**
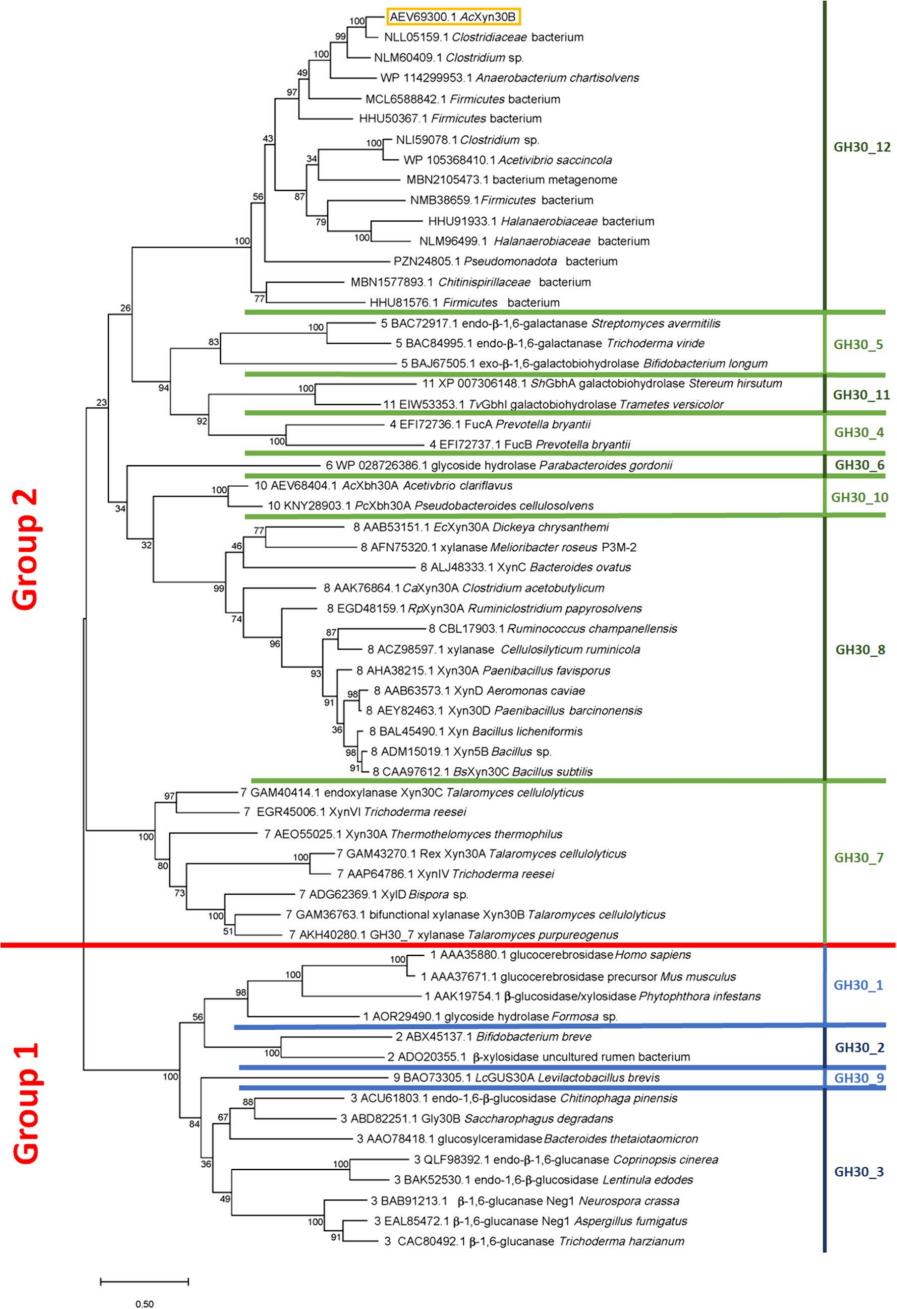
Phylogenetic relationship of GH30 characterized enzymes and the enzymes having similarity to *Ac*Xyn30B. The alignment performed using Clustal Omega was analyzed in MEGAX. The evolutionary history was inferred by using the Maximum Likelihood method and JTT matrix-based model. The percentage of replicate trees in which the associated taxa clustered together in the bootstrap test (500 replicates) are shown next to the branches

*Ac*Xyn30B (Clocl_2746, AEV69300.1, G8M2Z1) consists of 673 amino acids. The signal peptide is 28 amino acids long (SignalP) (Teufel et al. 2022) and the GH30 catalytic module (amino acids 29-446) is followed by a CBM6 module (amino acids 469-593), and a dockerin module (amino acids 602-673). A comparison of the full-length amino acid sequence to a protein database using the BlastP revealed that among the first 100 hits, there was only one characterized enzyme *Cp*Xyn30A from *Ruminiclostridium papyrosolvens* (WP_004618990.1, also containing CBM6 domain) (St John et al. 2014) which had 31.51% identity with *Ac*Xyn30B. The most similar proteins were uncharacterized GH30 proteins from the genera *Clostridium*, *Anaerobacterium*, and *Bacillota*. A pairwise sequence comparison of *Ac*Xyn30B catalytic domain with catalytic domains of several members of each GH30 subfamily showed that *Ac*Xyn30B is most similar to GH30_8 subfamily; however, the identity and similarity are quite low, about 24–27% and 38–44%, respectively. In the CAZy database, the enzyme is classified in the GH30 family but is not assigned to any subfamily. To reveal a relationship of *Ac*Xyn30B with other GH30 members, a phylogenetic tree was constructed (Fig. 1). Amino acid sequences of catalytic domains of characterized GH30 members from all known subfamilies and 14 amino acid sequences of proteins most similar to *Ac*Xyn30B according to BlastP search were initially aligned in Clustal Omega and the alignment was then analyzed in MEGAX. The phylogenetic tree (Fig. 1) shows that *Ac*Xyn30B and related proteins form a separate clade within group 2 (explained below) but clearly distinct from other subfamilies. Therefore, we propose *Ac*Xyn30B to be a founding member of a new GH30 subfamily, GH30_12.

The overall structure of GH30 enzymes is formed by (β/α)_8_ barrel which is linked to a side β-structure that is composed of 9 β-strands (Puchart et al. 2021). Based on the arrangement of the β_9_-domain relative to the (β/α)_8_ barrel, the GH30 family is divided into two groups: group 1 (subfamilies 1, 2, 3, 9) and group 2 (subfamilies 4, 5, 6, 7, 8, 10, 11) (St John et al. 2010). In group 1 the first three β-strands of the β_9_-domain precede the (β/α)_8_ barrel (Supplemental Fig. S1, highlighted in yellow) while in group 2 just one β-strand of the β_9_-domain is located in front of the (β/α)_8_ barrel. From the amino acid sequence alignment (Supplemental Fig. S1), it is obvious that in *Ac*Xyn30B, there is only one β-strand of the β_9_-domain preceding the (β/α)_8_ barrel, thus placing *Ac*Xyn30B into the group 2. Based on the sequence alignment Glu171 (an acid/base) and Glu279 (a nucleophile) are predicted to be catalytic residues (Supplemental Fig S1, highlighted in magenta). *Ac*Xyn30B and related enzymes do not contain a prokaryotic Arg which is responsible for glucuronoxylan specificity of the GH30_8 members (Supplemental Fig S1, highlighted in green). A different arginine residue, which often plays a similar role in eukaryotic GH30_7 glucuronoxylanases, is also absent in the sequence of *Ac*Xyn30B where Trp52 is found (Supplemental Fig S1, highlighted in gray). In this aspect, *Ac*Xyn30B resembles a non-specific GH30_7 xylanase *Tc*Xyn30C having Phe47 in the corresponding position (Nakamichi et al. 2020).

There are several differences in primary structure between GH30_7 and GH30_8 sequences (Puchart et al. 2021). The most obvious is the presence of much longer β2-α2 loop in the eukaryotic GH30_7 enzymes. The corresponding region of *Ac*Xyn30B is shorter, more similar to GH30_8 representatives (Supplemental Fig S1, highlighted in cyan). Moreover, the GH30_7 members contain additional short β-strands β8A and β8B in the β8-α8 segment. This region of *Ac*Xyn30B more resembles GH30_7 enzymes, but actually it is even longer (Supplemental Fig S1, highlighted in blue). It seems that in contrast to GH30_7 members, *Ac*Xyn30B does not lack the α6 helix (which is present in GH30_8 members), but the α7 helix is shorter and most similar in length to the non-specific GH30_8 enzymes *Cp*Xyn30A and *Ca*Xyn30A (Supplemental Fig. S1, highlighted in orange). Based on the primary structure comparison we can conclude that *Ac*Xyn30B and related enzymes have a special combination of structural features found in the enzymes from both GH30_7 and GH30_8 subfamilies.

### Catalytic properties

The activity of *Ac*Xyn30B was tested on different polysaccharides. The enzyme was not active on cellulose, hydroxyethyl cellulose, starch, laminarin (β-1,3-glucan with β-1,6 branches), and pustulan (β-1,6-glucan) but depolymerized glucuronoxylan (GX), rhodymenan (β-1,3-β-1,4-xylan, Rho), and arabinoxylan (AraX). The specific activity of *Ac*Xyn30B on GX, Rho, and AraX was quite low and very similar, 3.5, 2.9, and 2.7 U/mg, respectively. *Ac*Xyn30B exhibited slightly higher specific activities of 4.8 and 9.3 U/mg on chromogenic substrates NP-Xyl_2_ and NP-Xyl_3_, respectively. Similar activity on the polysaccharides and aryl glycosides of linear xylooligosaccharides indicates a wide substrate specificity, which is in contrast with narrow specificities of GH30 glucuronoxylanases and xylobiohydrolases and suggests that *Ac*Xyn30B is a non-specific xylanase. The differences in catalytic properties also support the phylogenetic classification of *Ac*Xyn30B into the separate clade. Kinetic parameters (Table 1) showed that GX is a little bit better substrate than AraX due to lower K_m_ value, and that the enzyme prefers longer oligosaccharides because the catalytic efficiency on NP-Xyl_3_ is higher than on NP-Xyl_2_.

**Table 1:** Kinetic parameters of *Ac*Xyn30B

Substrate	k_cat_ (s^−1^)	K_m_ (mM)	k_cat_/K_m_ (s^−1^.mM^−1^)
GX	4.6	1.1*	4.1*
AraX	4.5	2.8*	1.6*
NP-Xyl_2_	7.4	0.21	34.9
NP-Xyl_3_	10.4	0.23	56.1

*For GX and AraX, K_m_ and k_cat_/K_m_ are expressed in mg.ml^−1^ and s^−1^.mg^−1^.ml, respectively

**Fig. 2:**
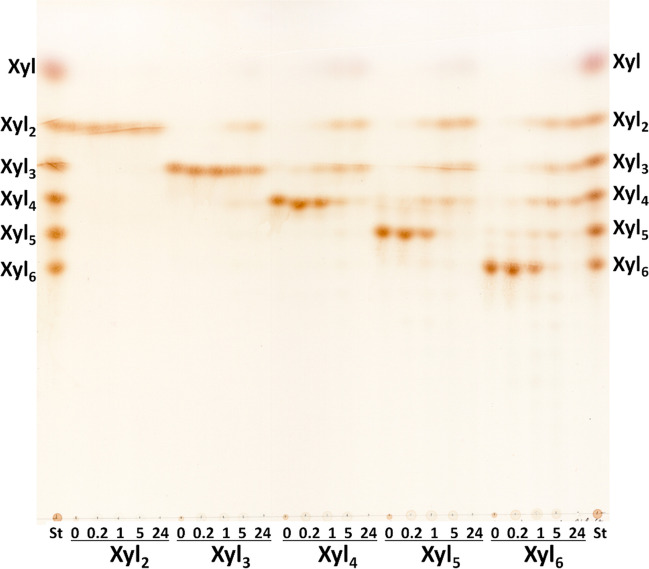
TLC analysis of the products formed from linear XOs (Xyl_2_ – Xyl_6_) by *Ac*Xyn30B after 10 min, 1 h, 5 h, and 24 h. St, standards of linear XOs

The activity of *Ac*Xyn30B was also qualitatively examined on linear xylooligosaccharides (XOs) Xyl_2_ – Xyl_6_ (Fig. 2). Xyl_2_ was not attacked by the enzyme while Xyl_3_ was slowly converted to Xyl and Xyl_2_. Xyl_4_ was hydrolyzed to approximately equal amount of Xyl, Xyl_2_, and Xyl_3_. The enzyme thus has no significant preference for binding the substrate in subsites -2 and +2 (generating Xyl_2_) over the subsites either −3 to +1 or −1 to +3 (yielding Xyl_3_ and Xyl). Xyl_2_ and Xyl_3_ were the major products formed from Xyl_5_, and Xyl_6_ was cleaved to Xyl_2_, Xyl_3_, and Xyl_4_, so in both cases, at least two subsites are occupied on both sides from the catalytic amino acids (from −2 to +2). Longer XOs seem to be hydrolyzed faster than shorter ones and at least four catalytic subsites need to be occupied for effective cleavage. In the 5-h hydrolysates, tiny amounts of XOs longer than a substrate were observed as a result of transglycosylation reaction.

**Fig. 3:**
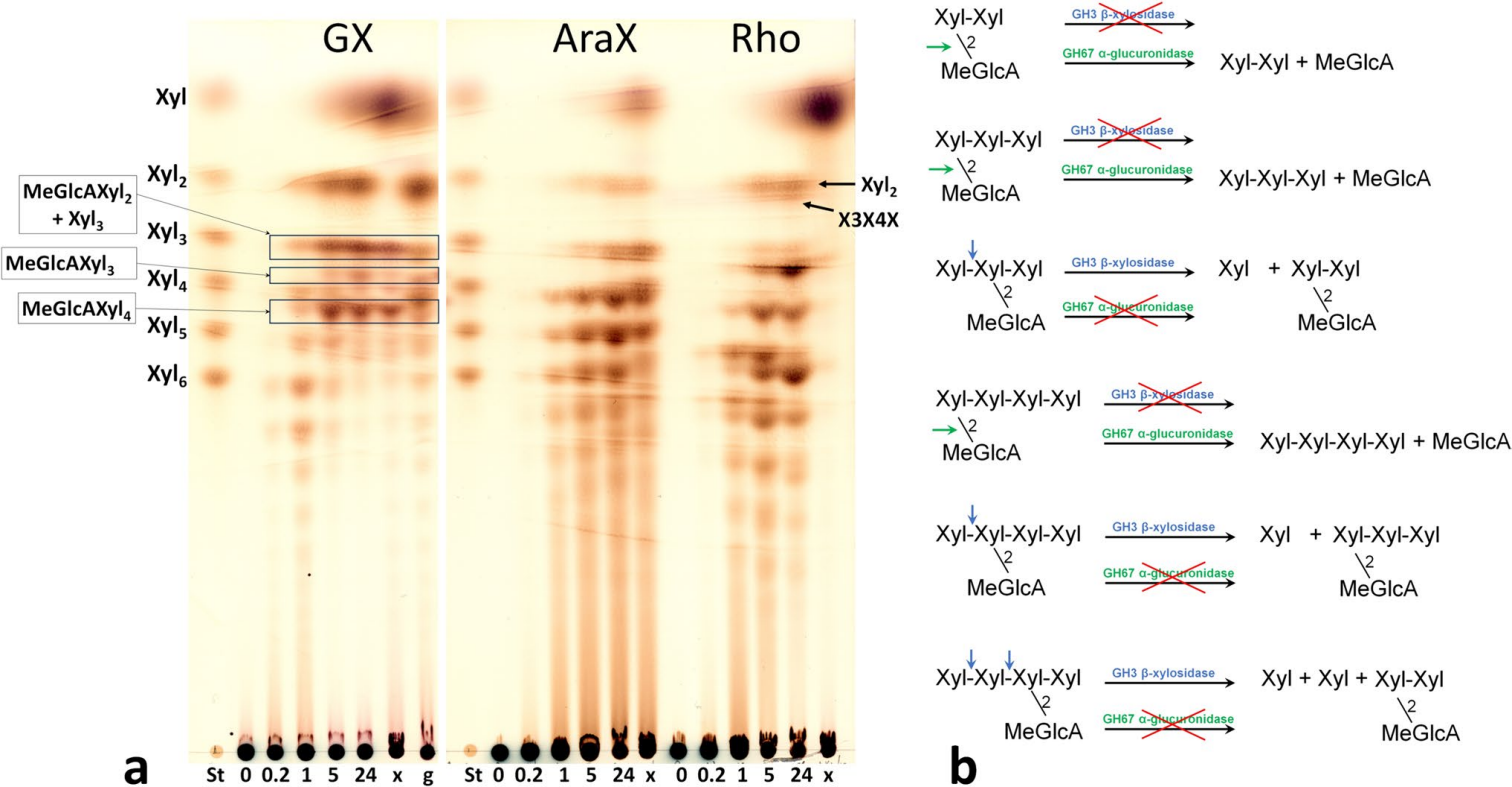
**a** TLC analysis of hydrolysis products released by *Ac*Xyn30B from beechwood glucuronoxylan (GX), wheat arabinoxylan (AraX) and rhodymenan (Rho) after 10 min, 1 h, 5 h, and 24 h, and after subsequent addition of either β-xylosidase (x) or α-glucuronidase (g). St, standards of linear XOs. **b** Action of β-xylosidase and α-glucuronidase on different acidic XOs which are produced from GX by *Ac*Xyn30B

**Fig. 4:**
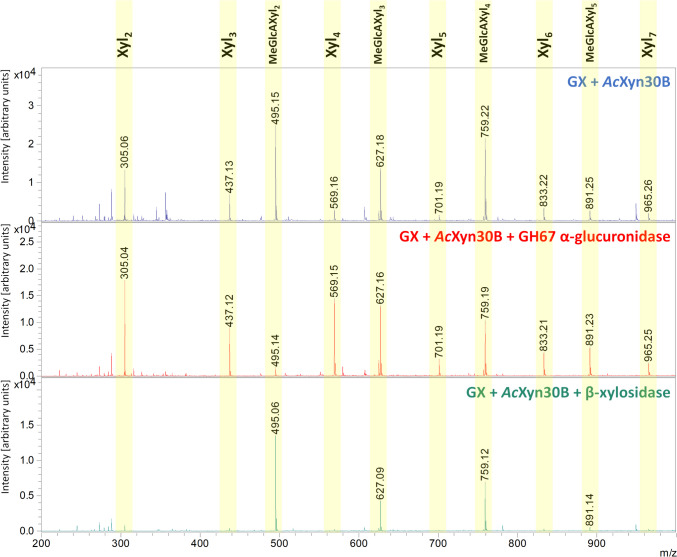
MALDI-TOF MS analysis of hydrolysis products released by *Ac*Xyn30B from beechwood glucuronoxylan after 24 h, and after subsequent addition of either α-glucuronidase or β-xylosidase

To reveal the mode of action of *Ac*Xyn30B on GX, AraX, and rhodymenan, the hydrolysates were analyzed by TLC (Fig. 3) and MALDI-TOF MS (Fig. 4). The detectable products of different lengths were produced from GX already after 10 min of hydrolysis. After 24 h they were shortened to linear oligosaccharides Xyl – Xyl_6_ and acidic XOs MeGlcAXyl_2_ – MeGlcAXyl_4_. To determine the structure of the released acidic XOs, either GH3 β-xylosidase or GH67 α-glucuronidase was applied to the 24-h hydrolysate (Figs. 3 and 4). The GH3 β-xylosidase is able to release non-substituted xylopyranosyl residue from the non-reducing end of XOs (Biely et al. 2016). α-Glucuronidases from GH67 family are known to release the (4-*O*-methyl-)glucuronic acid (GlcA/MeGlcA) only from the non-reducing end xylopyranosyl residue (Biely et al. 2016). The application of β-xylosidase on the GX hydrolysate did not affect the amount of MeGlcAXyl_2_, while the application of α-glucuronidase resulted in its disappearance accompanied with an increase in xylobiose amount (Figs. 3 and 4). This means that MeGlcA is attached to the non-reducing end xylose moiety of this acidic XO and its structure is MeGlcA^2^Xyl_2_. However, in the case of MeGlcAXyl_3_ and MeGlcAXyl_4_, the results were not so straightforward. Most of MeGlcAXyl_3_ was cleaved by β-xylosidase to MeGlcAXyl_2_ but a smaller part remained in the hydrolysate indicating that the predominant form of MeGlcAXyl_3_ is MeGlcA^2^Xyl_3,_ but MeGlcA^3^Xyl_3_ is also present. In contrast, most of MeGlcAXyl_4_ was hydrolyzed by α-glucuronidase to MeGlcA and Xyl_4_, meaning that MeGlcA^4^Xyl_4_ is the main MeGlcA-substituted Xyl_4_, but there are also other isomers (e.g., MeGlcA^3^Xyl_4_) present in the hydrolysate. To further inspect the hydrolysis of acidic XOs, *Ac*Xyn30B was applied on MeGlcA^3^Xyl_3_ and MeGlcA^3^Xyl_4_. After 24 h, MeGlcA^3^Xyl_3_ was not attacked, while MeGlcA^3^Xyl_4_ was hydrolyzed to MeGlcA^2^Xyl_3_ and Xyl. Prolonged incubation (3 days) led to a very slow cleavage of MeGlcA^3^Xyl_3_ to MeGlcA^2^Xyl_2_ and Xyl, while MeGlcA^2^Xyl_3_, a degradation product of MeGlcA^3^Xyl_4_, was slowly further converted to MeGlcA^2^Xyl_2_ (Supplemental Fig. S2).

**Fig. 5:**
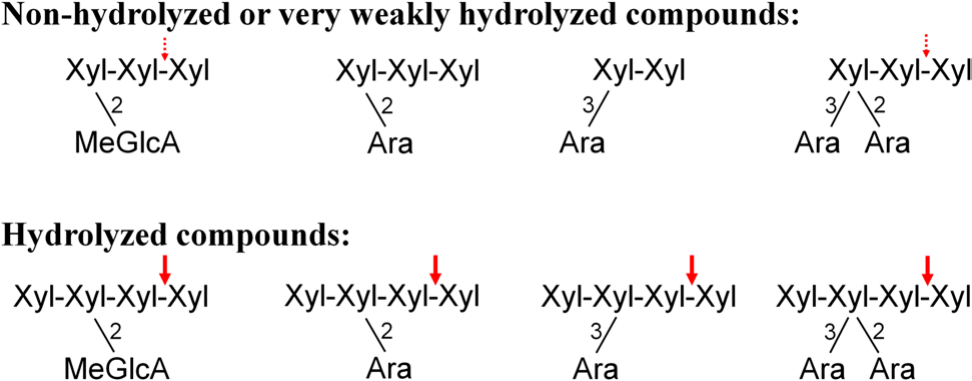
Various branched XOs tested as the substrates for *Ac*Xyn30B. The cleavage site is indicated by an arrow

AraX was hydrolyzed by *Ac*Xyn30B to a mixture of linear and Ara-substituted XOs which were difficult to identify (Fig. 3a). However, the mode of action of *Ac*Xyn30B on arabinosylated substrates can be assumed from the hydrolysis of short Ara-XOs of defined structure. After 24 h, the enzyme did not attack A^3^X, A^2^XX, and A^2+3^XX, but it released Xyl from XA^3^XX, XA^2^XX, and XA^2+3^XX (Fig. 5, Supplemental Fig. S3). When the mixtures were incubated for a prolonged time (3 days), a very low amount of Xyl was also liberated from A^2+3^XX (Supplemental Fig. S3). In all cases, Xyl was released from the reducing end of the substrates. This conclusion is based on experiments using the GH3 β-xylosidase or various α-arabinofuranosidases and is depicted and explained in Supplemental Fig. S4. The release of Xyl from the reducing end of XA^3^XX, XA^2^XX, and XA^2+3^XX means that these substrates are bound in the catalytic subsites −3, −2, −1, and +1 (cleavage occurring between the subsites −1 and +1). Singly or doubly Ara-substituted Xyl*p* residue is then accommodated in the −2 subsite and unsubstituted Xyl*p* residues are located in the subsites −3, −1, and +1. If we assume that one Xyl*p* shorter substrates (A^2^XX vs XA^2^XX and A^2+3^XX vs XA^2+3^XX) are accommodated in a similar way, they occupy the subsites −2, −1 and +1, but they are not cleaved or are cleaved very slowly. This indicates that the occupation of the −3 subsite by Xyl*p* residue promotes the hydrolysis of substituted XOs. In other words, substituted substrates are effectively cleaved only when at least four catalytic subsites of *Ac*Xyn30B are occupied (which is in consonance with hydrolysis of neutral and acidic XOs), Xyl*p* residue in the −1 subsite is non-substituted, the −3 subsite is occupied and the substituted Xyl*p* residue (at position 2 and/or 3) is located in the −2 subsite.

Hydrolysis of Rho by *Ac*Xyn30B yielded a mixture of β-1,4-linked and β-1,3-1,4-linked XOs (Fig. 3). Similarly to *Aa*Xyn30A, the enzyme was able to release small amount of isomeric xylotriose having the structure of β-d-Xyl*p*-1,3-β-d-Xyl*p*-1,4-β-d-Xyl (X3X4X) as the shortest mixed linkage oligosaccharide. However, the amount of longer XOs (degrees of polymerization (DP) 4-6) was higher, and β-1,3-1,4-linked XOs were prevailing.

## Discussion

Analysis of the *A. clariflavus* cellulosome has suggested that the GH30 enzymes (Clocl_1795, Clocl_2746) play a pivotal role in the *A. clariflavus* cellulosome as hemicellulases (Artzi et al. 2015). The recent discovery that Clocl_1795 is a xylobiohydrolase *Ac*Xbh30A and a founding member of the GH30_10 subfamily (Šuchová et al. 2021a; Crooks et al. 2021) prompted us to characterize the second GH30 xylanolytic enzyme *Ac*Xyn30B (Clocl_2746) of *A. clariflavus*. The protein had low amino acid sequence similarity to the characterized GH30 enzymes. Phylogenetic analysis revealed that *Ac*Xyn30B and related enzymes form a separate GH30_12 subfamily within group 2. Further inspection of *Ac*Xyn30B primary structure showed that the enzyme shares structural features of the enzymes from both GH30_7 and GH30_8 subfamilies. The shorter β2-α2 loop of *Ac*Xyn30B (similar to GH30_8 enzymes) can form less barriers for binding larger substrates to negative subsites, thus allowing a cleavage of the substrates by endo-mode. The presence of tryptophans in the positions corresponding to prokaryotic and eukaryotic arginines (that are responsible for glucuronoxylan specificity of GH30_8 and GH30_7 glucuronoxylanases) suggests that the catalytic site of *Ac*Xyn30B is paved by aromatic amino acids which allow non-specific binding of sugars. Due to the low similarity of *Ac*Xyn30B to other GH30 enzymes with solved 3-D structure, it was not possible to construct a reliable model of *Ac*Xyn30B and only its crystallization could shed more light on a role of particular amino acids in catalysis.

Similar specific activity of *Ac*Xyn30B on GX, AraX, and linear oligosaccharides indicate that *Ac*Xyn30B is a non-specific xylanase not preferring any substrate. There are two non-specific xylanases in the GH30_8 subfamily – *Cp*Xyn30A and *Ca*Xyn30A (St John et al. 2014, 2018), both lacking so-called prokaryotic arginine which is otherwise conserved in the GH30_8 members and responsible for their strict glucuronoxylanase specificity. While *Ca*Xyn30A was shown to prefer Ara-substituted substrates, the specific activity of *Cp*Xyn30A on GX, AraX, and Xyl_6_ was similar (St John et al. 2014). *Cp*Xyn30A did not hydrolyze Xyl_3_ and hydrolysis of Xyl_4_ was slow. The shortest acidic fragment released from GX by *Cp*Xyn30A was MeGlcAXyl_4_. On the contrary, *Ac*Xyn30B was able to slowly cleave Xyl_3_ and the shortest acidic fragment released from GX was MeGlcA^2^Xyl_2_ which means that *Ac*Xyn30B is able to cleave the substrates to shorter products than *Cp*Xyn30A. MeGlcA^2^Xyl_2_ was observed as the shortest acidic product released from GX also by non-specific xylanases from the GH30_7 subfamily – *Tc*Xyn30C and *Tl*Xyn30A (Nakamichi et al. 2019c; Šuchová et al. 2021c). Both enzymes initially hydrolyzed GX to a series of acidic products of the general structure MeGlcA^2^Xyl_n_ similarly to GH30 glucuronoxylanases. However, these acidic XOs were not accumulated in the reaction mixture but were subsequently converted to MeGlcA^2^Xyl_2_ and the corresponding linear XOs, which were finally converted to Xyl and Xyl_2_. *Ac*Xyn30B liberated the same final products, but also longer linear and acidic XOs remained in the hydrolysate after 24 h. MeGlcA^3^Xyl_3_, A^2^XX, and A^2+3^XX which are bound to 3 catalytic subsites only, were hardly hydrolyzed by *Ac*Xyn30B but all three compounds served as a substrate for *Tc*Xyn30C and *Tl*Xyn30A. This means that *Ac*Xyn30B requires at least 4 occupied catalytic subsites for effective cleavage while three occupied subsites are sufficient for the action of *Tc*Xyn30C and *Tl*Xyn30A, thus enabling the latter two enzymes to release shorter products. The presence of longer acidic XOs in the GX hydrolysate generated by *Ac*Xyn30B may be also the result of transglycosylation reactions which may occur in the presence of suitable acceptors. The variety of acidic XOs (carrying the MeGlcA substituent on different xylosyl residues) found in the 24-h hydrolysate of GX by *Ac*Xyn30B suggests that the enzyme initially does not attack the substrate as glucuronoxylanase but rather cleave GX non-specifically. MeGlcA-substituted Xyl*p* residue may be accommodated in several enzyme subsites except of the -1 subsite because none of the acidic XOs released from GX comprised MeGlcA-substituted Xyl*p* at the reducing end. On the other hand, MeGlcA^2^Xyl_2_ formed from GX indicates that decoration with MeGlcA is readily allowed in the subsites −2 and +1. It seems that main chain decoration reduces the enzyme action since *Ac*Xyn30B hydrolyses Xyl_4_ significantly faster than MeGlcA^3^Xyl_4_. In contrast, *Tc*Xyn30C and *Tl*Xyn30A were shown to prefer a cleavage of MeGlcA-substituted XOs (Nakamichi et al. 2019c; Šuchová et al. 2021c).

Microorganisms developed different strategies for a deconstruction of lignocellulosic material. In *A. clariflavus*, this process involves at least two cellulosomal xylanolytic GH30 enzymes: *Ac*Xbh30A and *Ac*Xyn30B. *Ac*Xbh30A is a xylobiohydrolase able to release prebiotic sugar xylobiose from glucuronoxylan and xylooligosaccharides, while highly substituted arabinoxylan is attacked only weakly (Šuchová et al. 2021a; Crooks et al. 2021). *Ac*Xyn30B is a non-specific xylanase hydrolyzing different types of xylan including arabinoxylan to shorter fragments. The phylogenetic analysis placed *Ac*Xyn30B to a new GH30_12 subfamily which pave the way for the discovery of more enzymes belonging to the same subfamily and having similar specificity in other microorganisms. The GH30 enzymes may cooperate with other well known xylanolytic enzymes what seems to be the case of *A. clariflavus* since additional predicted xylanases from GH10 and GH11 families were identified in its cellulosome complexes (Artzi et al. 2015). The presence of enzymes with different and complementary specificities enables the microorganism to hydrolyze a variety of materials available in the nature and compete with others. Although the non-specific GH30 xylanases and xylanases from families GH10 and GH11 attack the same substrates (e.g., glucuronoxylan and arabinoxylan), they release the products differing in length and branching. Therefore, they may act in synergy as was shown by GH30_8 glucuronoxylan-specific *Bs*XynC and GH11 xylanase A that are secreted simultaneously by *B. subtilis* (Rhee et al. 2014). Another cooperation of GH 30 xylanase (*Tt*Xyn30A) was found with lytic polysaccharide monooxygenase (LPMO) *Pc*AA14B from *Pycnoporus coccineus* operating on xylan polysaccharide(s) (Zerva et al. 2020). Although the LPMOs are not produced by bacteria, their synergistic action with other carbohydrate-active enzymes including xylanases of prokaryotic origin is of upmost importance from a view of their biotechnological exploitation because the oxidative enzymes typically work on complex insoluble substrates, which are much more resistant to hydrolytic enzymes. In this way, a saccharification of crude plant biomass may be significantly enhanced.

## Supplementary information

ESM 1 (PDF 510 kb)

## Data Availability

All data supporting the findings of this study are available within the paper and its Supplementary Information.

## References

[CR1] Artzi L, Morag E, Barak Y, Lamed R, Bayer EA (2015) *Clostridium clariflavum*: key cellulosome players are revealed by proteomic analysis. mBio 6(3):e00411–e00415. 10.1128/mBio.00411-15 25991683 10.1128/mBio.00411-15 PMC4442141

[CR2] Biely P, Hirsch J, la Grange DC, van Zyl WH, Prior BA (2000) A chromogenic substrate for a β-xylosidase-coupled assay of α-glucuronidase. Anal Biochem 286:289–294. 10.1006/abio.2000.4810 11067752 10.1006/abio.2000.4810

[CR3] Biely P, Puchart V, Stringer MA, Mørkeberg Krogh KBR (2014) *Trichoderma reesei* XYN VI – a novel appendage-dependent eukaryotic glucuronoxylan hydrolase. FEBS J 281:3894–3903. 10.1111/febs.12925 25041335 10.1111/febs.12925

[CR4] Biely P, Singh S, Puchart V (2016) Towards enzymatic breakdown of complex plant xylan structures: state of the art. Biotechnol Adv 34:1260–1274. 10.1016/j.biotechadv.2016.09.001 27620948 10.1016/j.biotechadv.2016.09.001

[CR5] Biely P, Vršanská M, Tenkanen M, Kluepfel D (1997) Endo-β-1,4-xylanase families: differences in catalytic properties. J Biotechnol 57:151–166. 10.1016/S0168-1656(97)00096-5 9335171 10.1016/s0168-1656(97)00096-5

[CR6] Crooks C, Bechle NJ, St John FJ (2021) A new subfamily of glycoside hydrolase family 30 with strict xylobiohydrolase function. Front Mol Biosci 8:714238. 10.3389/FMOLB.2021.714238 34557520 10.3389/fmolb.2021.714238 PMC8453022

[CR7] Drula E, Garron ML, Dogan S, Lombard V, Henrissat B, Terrapon N (2022) The carbohydrate-active enzyme database: functions and literature. Nucleic Acids Res 50:D571–D577. 10.1093/NAR/GKAB1045 34850161 10.1093/nar/gkab1045 PMC8728194

[CR8] Ebringerová A, Kramár A, Rendoš F, Domanský R (1967) Fractional extraction of hemicellulose from wood of hornbeam (*Carpinus betulus* L.). Holzforschung 21:74–77. 10.1515/hfsg.1967.21.3.74

[CR9] Felsenstein J (1985) Confidence limits on phylogenies: an approach using the bootstrap. Evolution 39:783–791. 10.2307/2408678 28561359 10.1111/j.1558-5646.1985.tb00420.x

[CR10] Jones DT, Taylor WR, Thornton JM (1992) The rapid generation of mutation data matrices from protein sequences. Bioinformatics 8:275–282. 10.1093/bioinformatics/8.3.275 10.1093/bioinformatics/8.3.275 1633570

[CR11] Katsimpouras C, Dedes G, Thomaidis NS, Topakas E (2019) A novel fungal GH30 xylanase with xylobiohydrolase auxiliary activity. Biotechnol Biofuels 12:120. 10.1186/S13068-019-1455-2 31110561 10.1186/s13068-019-1455-2 PMC6511221

[CR12] Kumar S, Stecher G, Li M, Knyaz C, Tamura K (2018) MEGA X: molecular evolutionary genetics analysis across computing platforms. Mol Biol Evol 35:1547–1549. 10.1093/molbev/msy096 29722887 10.1093/molbev/msy096 PMC5967553

[CR13] Li X, Kouzounis D, Kabel MA, de Vries RP, Dilokpimol A (2022) Glycoside hydrolase family 30 harbors fungal subfamilies with distinct polysaccharide specificities. N Biotechnol 67:32–41. 10.1016/j.nbt.2021.12.004 34952234 10.1016/j.nbt.2021.12.004

[CR14] Madeira F, Park YM, Lee J, Buso N, Gur T, Madhusoodanan N, Basutkar P, Tivey ARN, Potter SC, Finn RD, Lopez R (2019) The EMBL-EBI search and sequence analysis tools APIs in 2019. Nucleic Acids Res 47:W636–W641. 10.1093/NAR/GKZ268 30976793 10.1093/nar/gkz268 PMC6602479

[CR15] Nakamichi Y, Fouquet T, Ito S, Matsushika A, Inoue H (2019a) Mode of action of GH30-7 reducing-end xylose-releasing exoxylanase A (Xyn30A) from the filamentous fungus *Talaromyces cellulolyticus*. Appl Environ Microbiol 85:e00552–e00519. 10.1128/AEM.00552-19 31003983 10.1128/AEM.00552-19 PMC6581162

[CR16] Nakamichi Y, Fouquet T, Ito S, Watanabe M, Matsushika A, Inoue H (2019b) Structural and functional characterization of a bifunctional GH30-7 xylanase B from the filamentous fungus *Talaromyces cellulolyticus*. J Biol Chem 294:4065–4078. 10.1074/JBC.RA118.007207 30655295 10.1074/jbc.RA118.007207 PMC6422087

[CR17] Nakamichi Y, Fujii T, Fouquet T, Matsushika A, Inoue H (2019c) GH30-7 endoxylanase C from the filamentous fungus *Talaromyces cellulolyticus*. Appl Environ Microbiol 85:e01442–e01419. 10.1128/AEM.01442-19 31492671 10.1128/AEM.01442-19 PMC6821971

[CR18] Nakamichi Y, Fujii T, Watanabe M, Matsushika A, Inoue H (2020) Crystal structure of GH30-7 endoxylanase C from the filamentous fungus *Talaromyces cellulolyticus*. Acta Crystallogr F76:341–349. 10.1107/S2053230X20009024 10.1107/S2053230X20009024 PMC7397468 32744245

[CR19] Paleg LG (1959) Citric acid interference in estimation of reducing sugars with alkaline copper reagents. Anal Chem 31:1902–1904. 10.1021/ac60155a072

[CR20] Puchart V, Šuchová K, Biely P (2021) Xylanases of glycoside hydrolase family 30 – an overview. Biotechnol Adv 47:107704. 10.1016/J.BIOTECHADV.2021.107704 33548454 10.1016/j.biotechadv.2021.107704

[CR21] Rhee MS, Wei L, Sawhney N, Rice JD, St. John FJ, Hurlbert JC, Preston JF (2014) Engineering the xylan utilization system in *Bacillus subtilis* for production of acidic xylooligosaccharides. Appl Environ Microbiol 80:917–927. 10.1128/AEM.03246-13 24271172 10.1128/AEM.03246-13 PMC3911196

[CR22] Robert X, Gouet P (2014) Deciphering key features in protein structures with the new ENDscript server. Nucleic Acids Res 42:W320–W324. 10.1093/nar/gku316 24753421 10.1093/nar/gku316 PMC4086106

[CR23] Shiratori H, Sasaya K, Ohiwa H, Ikeno H, Ayame S, Kataoka N, Miya A, Beppu T, Ueda K (2009) *Clostridium clariflavum* sp. nov. and *Clostridium caenicola* sp. nov., moderately thermophilic, cellulose-/cellobiose-digesting bacteria isolated from methanogenic sludge. Int J Syst Evol Microbiol 59:1764–1770. 10.1099/ijs.0.003483-0 19542130 10.1099/ijs.0.003483-0

[CR24] St John FJ, Crooks C, Kim Y, Tan K, Joachimiak A (2022) The first crystal structure of a xylobiose-bound xylobiohydrolase with high functional specificity from the bacterial glycoside hydrolase family 30, subfamily 10. FEBS Lett 596:2449–2464. 10.1002/1873-3468.14454 35876256 10.1002/1873-3468.14454 PMC12279061

[CR25] St John FJ, Dietrich D, Crooks C, Balogun P, de Serrano V, Pozharski E, Smith JK, Bales E, Hurlbert J (2018) A plasmid borne, functionally novel glycoside hydrolase family 30 subfamily 8 endoxylanase from solventogenic *Clostridium*. Biochem J 475:1533–1551. 10.1042/BCJ20180050 29626157 10.1042/BCJ20180050 PMC5934979

[CR26] St John FJ, Dietrich D, Crooks C, Pozharski E, González JM, Bales E, Smith K, Hurlbert JC (2014) A novel member of glycoside hydrolase family 30 subfamily 8 with altered substrate specificity. Acta Crystallogr D Biol Crystallogr 70:2950–2958. 10.1107/S1399004714019531 25372685 10.1107/S1399004714019531 PMC4722856

[CR27] St John FJ, González JM, Pozharski E (2010) Consolidation of glycosyl hydrolase family 30: a dual domain 4/7 hydrolase family consisting of two structurally distinct groups. FEBS Lett 584:4435–4441. 10.1016/j.febslet.2010.09.051 20932833 10.1016/j.febslet.2010.09.051

[CR28] St. John FJ, Rice JD, Preston JF (2006) Characterization of XynC from *Bacillus subtilis* subsp. *subtilis* strain 168 and analysis of its role in depolymerization of glucuronoxylan. J Bacteriol 188:8617–8626. 10.1128/JB.01283-06 17028274 10.1128/JB.01283-06 PMC1698249

[CR29] Šuchová K, Puchart V, Biely P (2021a) A novel bacterial GH30 xylobiohydrolase from *Hungateiclostridium clariflavum*. Appl Microbiol Biotechnol 105:185–195. 10.1007/S00253-020-11023-X 33215261 10.1007/s00253-020-11023-x

[CR30] Šuchová K, Puchart V, Spodsberg N, Mørkeberg Krogh KBR, Biely P (2020) A novel GH30 xylobiohydrolase from *Acremonium alcalophilum* releasing xylobiose from the non-reducing end. Enzyme Microb Technol 134:109484. 10.1016/J.ENZMICTEC.2019.109484 32044031 10.1016/j.enzmictec.2019.109484

[CR31] Šuchová K, Puchart V, Spodsberg N, Mørkeberg Krogh KBR, Biely P (2021b) Catalytic diversity of GH30 xylanases. Molecules 26:4528. 10.3390/MOLECULES26154528 34361682 10.3390/molecules26154528 PMC8347883

[CR32] Šuchová K, Spodsberg N, Mørkeberg Krogh KBR, Biely P, Puchart V (2021c) Non-specific GH30_7 endo-β-1,4-xylanase from *Talaromyces leycettanus*. Molecules 26:4614. 10.3390/MOLECULES26154614 34361767 10.3390/molecules26154614 PMC8347862

[CR33] Tenkanen M, Vršanská M, Siika-Aho M, Wong DW, Puchart V, Penttilä M, Saloheimo M, Biely P (2013) Xylanase XYN IV from *Trichoderma reesei* showing exo- and endo-xylanase activity. FEBS J 280:285–301. 10.1111/FEBS.12069 23167779 10.1111/febs.12069

[CR34] Teufel F, Almagro Armenteros JJ, Johansen AR, Gíslason MH, Pihl SI, Tsirigos KD, Winther O, Brunak S, von Heijne G, Nielsen H (2022) SignalP 6.0 predicts all five types of signal peptides using protein language models. Nat Biotechnol 40:1023–1025. 10.1038/s41587-021-01156-3 34980915 10.1038/s41587-021-01156-3 PMC9287161

[CR35] Vršanská M, Kolenová K, Puchart V, Biely P (2007) Mode of action of glycoside hydrolase family 5 glucuronoxylan xylanohydrolase from *Erwinia chrysanthemi*. FEBS J 274:1666–1677. 10.1111/j.1742-4658.2007.05710.x 17381510 10.1111/j.1742-4658.2007.05710.x

[CR36] Zerva A, Pentari C, Grisel S, Berrin J-G, Topakas E (2020) A new synergistic relationship between xylan-active LPMO and xylobiohydrolase to tackle recalcitrant xylan. Biotechnol Biofuels 13:142. 10.1186/s13068-020-01777-x 32793303 10.1186/s13068-020-01777-x PMC7419196

