# Supplementary material for: Characterization of a novel GH30 non-specific endoxylanase AcXyn30B from Acetivibrio clariflavus
Source: Appl Microbiol Biotechnol. 2024 Apr 29;108(1):312. doi: 10.1007/s00253-024-13155-w (PMC11058611; doi:10.1007/s00253-024-13155-w)
Supplement: Supplementary file 1 — (PDF 510 kb) [file 253_2024_13155_MOESM1_ESM.pdf]

Applied Microbiology and Biotechnology

Supplementary Material

Characterization of a novel GH30 non-specific endoxylanase *Ac*Xyn30B from *Acetivibrio clariflavus*

Katarína Šuchová[1*], Walid Fathallah[1,2], Vladimír Puchart[1]

[1]Institute of Chemistry, Slovak Academy of Sciences, Dúbravská cesta 9, 845 38 Bratislava, Slovakia
[2]Faculty of Science, Beni-Suef University, 625 11 Beni-Suef, Egypt

*Corresponding author: Katarína Šuchová, E-mail: Katarina.Suchova@savba.sk

```
GH30_3_AAO78418.1

GH30_3_AAO78418.1         1   .........................................minmrnialtflg.....c
GH30_1_AAA35880.1         1   mefsspsreecpkplsrvsimagsltgllllqavswasgARPCIPKSFGYSSVV.....C
GH30_2_ABX45137.1             ............................................................
GH30_9_LcGUS30            1   ............................................MKKFQHEF........
GH30_11_ShGbhA            1   ........................................ml.spphllsssva....ta
GH30_5_BAC84995.1         1   ............................................mrsivlpsla......
GH30_4_PbFucA             1   ............................................mkrilpslff......
GH30_HHU50367.1           1   ............................................mkrqyvlplvmvivgl
GH30_WP_114299953.1       1   ..........................................mkkgkililpvil....a
GH30_NLM60409.1           1   ..........................................mkirsklslllil....t
GH30_AcXyn30B             1   ..........................................mlkkrklvlsvvl....s
GH30_NLL05159.1           1   ..........................................mvmkkklalsvvm....s
GH30_7_TcXyn30A           1   ............................................mrhpip..il......
GH30_7_TcXyn30B           1   ............................................mvfskv.ava......
GH30_7_TcXyn30C           1   ............................................mwslksnsta......
GH30_7_TrXynVI            1   ............................................mialvp..fv......
GH30_6_WP_028726386.1     1   ..........................................mktrrnltlglf....ac
GH30_10_AcXbh30A          1   ............................................mrktvslttvaallis
GH30_8_EcXyn30A           1   ........................................mng....nvs.lwvrhclha
GH30_8_CaXyn30A           1   ........................................mniklkrtl.islvafsmtc
GH30_8_BsXyn30C           1   ........................................miprikkticvllvcftm.l
GH30_8_CpXyn30A           1   ........................................mfknmkktiskvlvssiims
GH30_8_CpXyn30A
```

```
                                                      β1X        β2X
GH30_3_AAO78418.1

GH30_3_AAO78418.1        15   ftilaACSNSDDAEKPVVPVPTGDVAIYTTTSSLTRDLTRDAV.NFSPKDNLAPTTITLN
GH30_1_AAA35880.1        56   VCNATYCDSFD...PPTFPAL.GTFSRYESTRS..GRRMELSMGPIQANHTGTGLLLTLQ
GH30_2_ABX45137.1         1   ..............MTTWIATTQDDK............LADRT.SDIESTNATTAADLQL
GH30_9_LcGUS30            9   .................WTATSGDL.........TQRRTPLAT.PDYQSTSAAAVKIIID
GH30_11_ShGbhA           16   lsils......................................gilapqh.vta..ATVS
GH30_5_BAC84995.1        11   ...........................................lalfsqraraDTTLTID
GH30_4_PbFucA            11   ...as.......................................lcmgisaqsntKVTIN
GH30_HHU50367.1          17   lvvlg......................................favvpqtnaVTYNASVN
GH30_WP_114299953.1      15   vimts......................................fisvpnvsaAAYSVSVD
GH30_NLM60409.1          15   lvm.t......................................cllnvqsgaANYNVSVD
GH30_AcXyn30B            15   mvl.t......................................lvnfpktnaaSYTASVD
GH30_NLL05159.1          15   iil.s......................................fmifpktdaASYTASVD
GH30_7_TcXyn30A           9   .avlg.....................................rasaWSYSQTLSANIQVN
GH30_7_TcXyn30B          10   .asal.....................................s....lgayaidaQINVD
GH30_7_TcXyn30C          11   .lava.....................................l....ngivalgQTITVN
GH30_7_TrXynVI            9   .iiag.....................................a....atlsvaQTTITVN
GH30_6_WP_028726386.1    15   lfllgsCTDNEFA..........................DQKAGSSIATKAVANTDVVLH
GH30_10_AcXbh30A         17   mlil.......................................v..lptnlyaASTVTVD
GH30_8_EcXyn30A          16   alfv.......................................satagsfsvyaDTVKID
GH30_8_CaXyn30A          20   lpfv.......................................gt.gssvkaASNDATIN
GH30_8_BsXyn30C          20   svml.......................................gp.gatevlaASDVTVN
GH30_8_CpXyn30A          21   alfm.......................................vs.apagvsaASDVTVN
GH30_8_CpXyn30A
```

```
                              β3X       β1                    α1
GH30_3_AAO78418.1

GH30_3_AAO78418.1        74   PAEQYQTMDGFGAAITGSTCYNLLLM..KPADRHAFLTETFSDK.D.........GFGFS
GH30_1_AAA35880.1       110   PEQKFQKVKGFGGAMTDAAALNILAL..SP.PAQNLLLKSYFSE.E.........GIGYN
GH30_2_ABX45137.1        34   DGNEYQALRGFGGCFNELGWLPLQNV...SEAERDQIIKELFSP.D.........EMNFT
GH30_9_LcGUS30           42   PADRHQPWLGGGAAITDSAAYLLWSV..MSAEQRRALLTELFDP.D.........QGGFS
GH30_11_ShGbhA           35   .SEVAQTFLGIGGSG..AWWPTDLYLYP.DEVKKNLSA.LLFSE.D.........GMGIS
GH30_5_BAC84995.1        28   PTSNWGTWEGWGVSL..AWWAKAFGN......RDDLAS.VFFSRNNQAVNGQTLPGLGFN
GH30_4_PbFucA            29   PSKTYQTMRSFGASD..CWTAEYVAQYFSDTQRQNAARWLFSSEVDA...QGNPLGIALS
GH30_HHU50367.1          39   VNTTYQTLEGFGASI..AWYQSYLTA...HPNREEVIDLLFKD.............LGID
GH30_WP_114299953.1      37   INTSYQTLEGFGAAI..AWSNESLTE...HPNRAGLYKTLFFD.............SGLD
GH30_NLM60409.1          36   INTKYQTLEGFGAAI..AWYNNWVTG...HPNKNELYNVLFYD.............LGLD
GH30_AcXyn30B            36   VNTTYQTLEGFGAAI..AWYNDYLTT...HPNKNDLYRILFYD.............SGLD
GH30_NLL05159.1          36   INTTYQTLEGFGAAI..AWYNDYLTT...HPNKNDLYKILFYD.............SGLD
GH30_7_TcXyn30A          31   ALQRYQEMIGGGCSGAFGWACQQFPTTGLTPENQEEVTKILFDENI.........G.GLS
GH30_7_TcXyn30B          28   LQARYQSVDGFGCSQAFQRAEDIFGKYGLSPKNQSYVLDLMYSEER.........GAGFT
GH30_7_TcXyn30C          29   PSTTYQTIDGFGFSEAFGFGAPIASA...SASIQTQVTNYLFSTTT.........GAGLT
GH30_7_TrXynVI           27   VGSKFQQIDGFGFSQAFGRAREFQSA...NATAQKQALDLLFSTST.........GAGFS
GH30_6_WP_028726386.1    49   WDKEEQNIDGFGVAQ.AGWADYLYA....HRKRDTVLDLMF..GKD.........GLHLN
GH30_10_AcXbh30A         36   WDTTYQTIDGFGVSEAFHQSNNIARL...GETKQNEIYDLLFSTTD.........GAGFS
GH30_8_EcXyn30A          37   ANVNYQIIQGFGGMSGVGWI.........NDLTTEQINTAYGSGVG.........QIGLS
GH30_8_CaXyn30A          40   VAAKHQTIRGFGASS..AWC.........GALSDTCMDTLY....K.........NAGLD
GH30_8_BsXyn30C          40   VSAEKQVIRGFGGMNHPAWA.........GDLTAAQRETAFGNGQN.........QLGFS
GH30_8_CpXyn30A          41   LGSTKQEIRGFGASS..AWC.........GTISDYVMNSLY....G.........DLGYS
GH30_8_CpXyn30A
                              β1X       β1                       α1
```

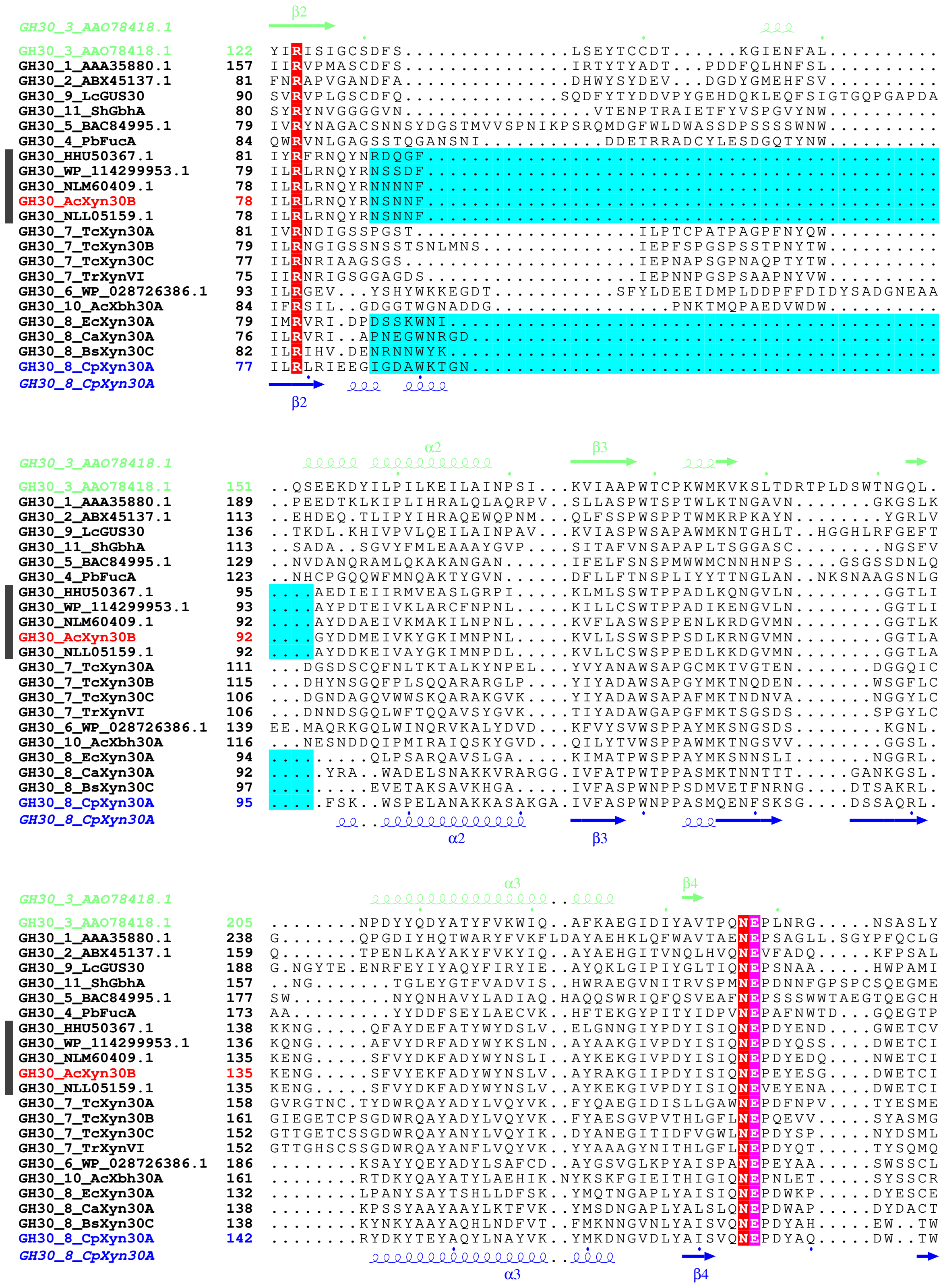
GH30_3_AAO78418.1 β2
GH30_3_AAO78418.1 122 YIRISIGCSDFS..............LSEYTCCDT......KGIENFAL..........
GH30_1_AAA35880.1 157 IIRVPMASCDFS..............IRTYTYADT...PDDFQLHNFSL..........
GH30_2_ABX45137.1 81 FNRAPVGANDFA..............DHWYSYDEV...DGDYGMEHFSV..........
GH30_9_LcGUS30 90 SVRVPLGSCDFQ.............SQDFYTYDDVPYGEHDQKLEQFSIGTGQPGAPDA
GH30_11_ShGbhA 80 SYRYNVGGGGVN................VTENPTRAIETFYVSPGVYNW..........
GH30_5_BAC84995.1 79 IVRYNAGACSNNSYDGSTMVVSPNIKPSRQMDGFWLDWASSDPSSSSWNW..........
GH30_4_PbFucA 84 QWRVNLGAGSSTQGANSNI...........DDETRRADCYLESDGQTYNW..........
GH30_HHU50367.1 81 IYRFRNQYNRDQGF...
GH30_WP_114299953.1 79 ILRLRNQYRNSSDF...
GH30_NLM60409.1 78 ILRLRNQYRNNNNF...
GH30_AcXyn30B 78 ILRLRNQYRNSNNF...
GH30_NLL05159.1 78 ILRLRNQYRNSNNF...
GH30_7_TcXyn30A 81 IVRNDIGSSPGST...................ILPTCPATPAGPFNYQW..........
GH30_7_TcXyn30B 79 ILRNGIGSSNSSTSNLMNS...............IEPFSPGSPSSTPNYTW..........
GH30_7_TcXyn30C 77 ILRNRIAAGSGS.....................IEPNAPSGPNAQPTYTW..........
GH30_7_TrXynVI 75 IIRNRIGSGGAGDS..................IEPNNPGSPSAAPNYVW..........
GH30_6_WP_028726386.1 93 ILRGEV...YSHYWKKEGDT...........SFYLDEEIDMPLDDPFFDIDYSADGNEAA
GH30_10_AcXbh30A 84 IFRSIL..GDGGTWGNADDG...............PNKTMQPAEDVWDW..........
GH30_8_EcXyn30A 79 IMRVRI.DPDSSKWNI...
GH30_8_CaXyn30A 76 ILRVRI..APNEGWNRGD...
GH30_8_BsXyn30C 82 ILRIHV.DENRNNWYK...
GH30_8_CpXyn30A 77 ILRLRIEEGIGDAWKTGN...
GH30_8_CpXyn30A
β2
GH30_3_AAO78418.1 α2 β3
GH30_3_AAO78418.1 151 ..QSEEKDYILPILKEILAINPSI...KVIAAPWTCPKWMKVKSLTDRTPLDSWTNGQL.
GH30_1_AAA35880.1 189 ..PEEDTKLKIPLIHRALQLAQRPV..SLLASPWTSPTWLKTNGAVN.......GKGSLK
GH30_2_ABX45137.1 113 ..EHDEQ.TLIPYIHRAQEWQPNM...QLFSSPWSPPTWMKRPKAYN........YGRLV
GH30_9_LcGUS30 136 ..TKDL.KHIVPVLQEILAINPAV...KVIASPWSAPAWMKNTGHLT..HGGHLRFGEFT
GH30_11_ShGbhA 113 ..SADA..SGVYFMLEAAAYGVP....SITAFVNSAPAPLTSGGASC........NGSFV
GH30_5_BAC84995.1 129 ..NVDANQRAMLQKAKANGAN......IFELFSNSPMWWMCNNHNPS....GSGSSDNLQ
GH30_4_PbFucA 123 ..NHCPGQQWFMNQAKTYGVN......DFLLFTNSPLIYYTTNGLAN..NKSNAAGSNLG
GH30_HHU50367.1 95 ....AEDIEIIRMVEASLGRPI.....KLMLSSWTPPADLKQNGVLN........GGTLI
GH30_WP_114299953.1 93 ....AYPDTEIVKLARCFNPNL.....KILLCSWTPPADIKENGVLN........GGTLI
GH30_NLM60409.1 92 ....AYDDAEIVKMAKILNPNL.....KVFLASWSPPENLKRDGVMN........GGTLK
GH30_AcXyn30B 92 ....GYDDMEIVKYGKIMNPNL.....KVLLSSWSPPSDLKRNGVMN........GGTLA
GH30_NLL05159.1 92 ....AYDDKEIVAYGKIMNPDL.....KVLLCSWSPPEDLKKDGVMN........GGTLA
GH30_7_TcXyn30A 111 ...DGSDSCQFNLTKTALKYNPEL...YVYANAWSAPGCMKTVGTEN.......DGGQIC
GH30_7_TcXyn30B 115 ...DHYNSGQFPLSQQARARGLP....YIYADAWSAPGYMKTNQDEN.......WSGFLC
GH30_7_TcXyn30C 106 ...DGNDAGQVVWWSKQARAKGVK...YIYADAWSAPAFMKTNDNVA.......NGGYLC
GH30_7_TrXynVI 106 ...DNNDSGQLWFTQQAVSYGVK....TIYADAWGAPGFMKTSGSDS.......SPGYLC
GH30_6_WP_028726386.1 139 EE.MAQRKGQLWINQRVKALYDVD...KFVYSVWSPPAYMKSNGSDS........KGNL.
GH30_10_AcXbh30A 116 ...NESNDDQIPMIRAIQSKYGVD...QILYTVWSPPAWMKTNGSVV........GGSL.
GH30_8_EcXyn30A 94 .........QLPSARQAVSLGA.....KIMATPWSPPAYMKSNNSLI......NGGRL.
GH30_8_CaXyn30A 92 ....YRA..WADELSNAKKVRARGG.IVFATPWTPPASMKTNNTTT......GANKGSL.
GH30_8_BsXyn30C 97 .........EVETAKSAVKHGA.....IVFASPWNPPSDMVETFNRNG....DTSAKRL.
GH30_8_CpXyn30A 95 ....FSK..WSPELANAKKASAKGA.IVFASPWNPPASMQENFSKSG....DSSAQRL.
GH30_8_CpXyn30A
α2 β3
GH30_3_AAO78418.1 α3 β4
GH30_3_AAO78418.1 205 .......NPDYYQDYATYFVKWIQ..AFKAEGIDIYAVTPQNEPLNRG.....NSASLY
GH30_1_AAA35880.1 238 G.......QPGDIYHQTWARYFVKFLDAYAEHKLQFWAVTAENEPSAGLL.SGYPFQCLG
GH30_2_ABX45137.1 159 Q.......TPENLKAYAKYFVKYIQ..AYAEHGITVNQLHVQNEVFADQ.....KFPSAL
GH30_9_LcGUS30 188 G.NGYTE.ENRFEYIYAQYFIRYIE..AYQKLGIPIYGLTIQNEPSNAA.....HWPAMI
GH30_11_ShGbhA 157 ..NG.......TGLEYGTFVADVIS..HWRAEGVNITRVSPMNEPDNNFGPSPCSQEGME
GH30_5_BAC84995.1 177 SW........NYQNHAVYLADIAQ.HAQQSWRIQFQSVEAFNEPSSSWWTAEGTQEGCH
GH30_4_PbFucA 173 AA.........YYDDFSEYLAECVK..HFTEKGYPITYIDPVNEPAFNWTD...GQEGTP
GH30_HHU50367.1 138 KKNG.....QFAYDEFATYWYDSLV..ELGNNGIYPDYISIQNEPDYEND....GWETCV
GH30_WP_114299953.1 136 KQNG.....AFVYDRFADYWYKSLN..AYAAKGIVPDYISIQNEPDYQSS....DWETCI
GH30_NLM60409.1 135 KENG.....SFVYDKFADYWYNSLI..AYKEKGIVPDYISIQNEPDYEDQ....NWETCI
GH30_AcXyn30B 135 KENG.....SFVYEKFADYWYNSLV..AYRAKGIIPDYISIQNEPEYESG....DWETCI
GH30_NLL05159.1 135 KENG.....SFVYDKFADYWYNSLV..AYKEKGIVPDYISIQNEVEYENA....DWETCI
GH30_7_TcXyn30A 158 GVRGTNC.TYDWRQAYADYLVQYVK..FYQAEGIDISLLGAWNEPDFNPV....TYESME
GH30_7_TcXyn30B 161 GIEGETCPSGDWRQAYADYLVQYVK..FYAESGVPVTHLGFLNEPQEVV.....SYASMG
GH30_7_TcXyn30C 152 GTTGETCSSGDWRQAYANYLVQYIK..DYANEGITIDFVGWLNEPDYSP.....NYDSML
GH30_7_TrXynVI 152 GTTGHSCSSGDWRQAYANFLVQYVK..YYAAAGYNITHLGFLNEPDYQT.....TYSQMQ
GH30_6_WP_028726386.1 186 ........KSAYYQEYADYLSAFCD..AYGSVGLKPYAISPANEPEYAA.....SWSSCL
GH30_10_AcXbh30A 161 ........RTDKYQAYATYLAEHIK.NYKSKFGIEITHIGIQNEPNLET.....SYSSCR
GH30_8_EcXyn30A 132 ........LPANYSAYTSHLLDFSK..YMQTNGAPLYAISIQNEPDWKP.....DYESCE
GH30_8_CaXyn30A 138 ........KPSSYAAYAAYLKTFVK..YMSDNGAPLYALSLQNEPDWAP.....DYDACT
GH30_8_BsXyn30C 138 ........KYNKYAAYAQHLNDFVT..FMKNNGVNLYAISVQNEPDYAH.....EW..TW
GH30_8_CpXyn30A 142 ........RYDKYTEYAQYLNAYVK..YMKDNGVDLYAISVQNEPDYAQ.....DW..TW
GH30_8_CpXyn30A
α3 β4

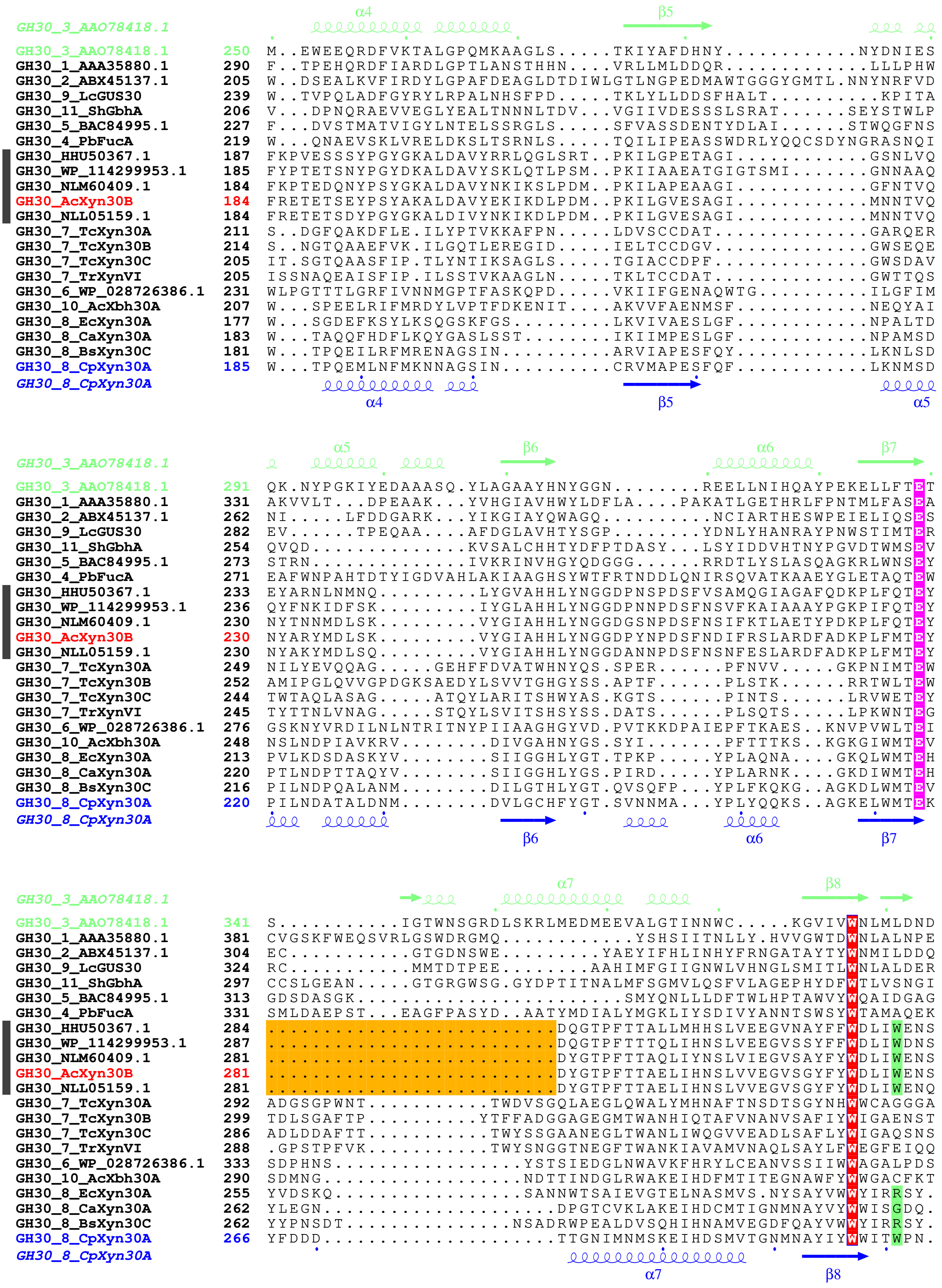
GH30_3_AAO78418.1
α4 β5
GH30_3_AAO78418.1 250 M..EWEEQRDFVKTALGPQMKAAGLS.....TKIYAFDHNY...........NYDNIES
GH30_1_AAA35880.1 290 F..TPEHQRDFIARDLGPTLANSTHHN....VRLLMLDDQR............LLLPHW
GH30_2_ABX45137.1 205 W..DSEALKVFIRDYLGPAFDEAGLDTDIWLGTLNGPEDMAWTGGGYGMTL.NNYNRFVD
GH30_9_LcGUS30 239 W..TVPQLADFGYRYLRPALNHSFPD.....TKLYLLDDSFHALT.........KPITA
GH30_11_ShGbhA 206 V..DPNQRAEVVEGLYEALTNNNLTDV...VGIIVDESSSLSRAT......SEYSTWLP
GH30_5_BAC84995.1 227 F...DVSTMATVIGYLNTELSSRGLS.....SFVASSDENTYDLAI......STWQGFNS
GH30_4_PbFucA 219 W...QNAEVSKLVRELDKSLTSRNLS.....TQILIPEASSWDRLYQQCSDYNGRASNQI
GH30_HHU50367.1 187 FKPVESSSYPGYGKALDAVYRRLQGLSRT.PKILGPETAGI...........GSNLVQ
GH30_WP_114299953.1 185 FYPTETSNYPGYDKALDAVYSKLQTLPSM..PKIIAAEATGIGTSMI.......GNNAAQ
GH30_NLM60409.1 184 FKPTEDQNYPSYGKALDAVYNKIKSLPDM..PKILAPEAAGI...........GFNTVQ
GH30_AcXyn30B 184 FRETETSEYPSYAKALDAVYEKIKDLPDM..PKILGVESAGI...........MNNTVQ
GH30_NLL05159.1 184 FRETETSDYPGYGKALDIVYNKIKDLPDM..PKILGVESAGI...........MNNTVQ
GH30_7_TcXyn30A 211 S..DGFQAKDFLE.ILYPTVKKAFPN.....LDVSCCDAT............GARQER
GH30_7_TcXyn30B 214 S..NGTQAAEFVK.ILGQTLEREGID.....IELTCCDGV............GWSEQE
GH30_7_TcXyn30C 205 IT.SGTQAASFIP.TLYNTIKSAGLS.....TGIACCDPF............GWSDAV
GH30_7_TrXynVI 205 ISSNAQEAISFIP.ILSSGTVKAAGLN.....TKLTCCDAT............GWTTQS
GH30_6_WP_028726386.1 231 WLPGTTTLGRFIVNNMGPTFASKQPD.....TQILIFGENAQWTG.........ILGFIM
GH30_10_AcXbh30A 207 W..SPEELRIFMRDYLVPTFDKENIT....AKVVFAENMSF............NEQYAI
GH30_8_EcXyn30A 177 W...SGDEFKSYLKSQGSKFGS.........LKVIVAESLGF............NPALTD
GH30_8_CaXyn30A 183 W...TAQQFHDFLKQYGASLSST........IKIIMPESLGF............NPAMSD
GH30_8_BsXyn30C 181 W...TPQEILRFMRENAGSIN..........ARVIAPESFQY............LKNLSD
GH30_8_CpXyn30A 185 W...TPQEMLNFMKNNAGSIN..........CRVMAPESFQF............LKNMSD
GH30_8_CpXyn30A
α4 β5 α5
GH30_3_AAO78418.1
α5 β6 α6 β7
GH30_3_AAO78418.1 291 QK.NYPGKIYEDAAASQ.YLAGAAYHNYGGN........REELLNIHQAYPEKELLFTET
GH30_1_AAA35880.1 331 AKVVLT...DPEAAK...YVHGIAVHWYLDFLA....PAKATLGETHRLFPNTMLFASEA
GH30_2_ABX45137.1 262 NI.....LFDDGARK...YIKGIAYQWAGQ.........NCIARTHESWPEIELIQSES
GH30_9_LcGUS30 282 EV......TPEQAA....AFDGLAVHTYSGP.........YDNLYHANRAYPNWSTIMTER
GH30_11_ShGbhA 254 QVQD.............KVSALCHHTYDFPTDASY...LSYIDDVHTNYPGVDTWMSEV
GH30_5_BAC84995.1 273 STRN.............IVKRINVHGYQDGGG......RRDTLYSLASQAGKRLWNSEY
GH30_4_PbFucA 271 EAFWNPAHTDTYIGDVAHLAKIAAGHSYWTFRTNDDLQNIRSQVATKAAEYGLETAQTEW
GH30_HHU50367.1 233 EYARNLNMNQ.........LYGVAHHLYNGGDPNSPDSFVSAMQGIAGAFQDKPLFQTEY
GH30_WP_114299953.1 236 QYFNKIDFSK.........IYGLAHHLYNGGDPNNPDSFNSVFKAIAAAYPGKPIFQTEY
GH30_NLM60409.1 230 NYTNNMDLSK.........VYGIAHHLYNGGDGSNPDSFNSIFKTLAETYPDKPLFQTEY
GH30_AcXyn30B 230 NYARYMDLSK.........VYGIAHHLYNGGDPYNPDSFNDIFRSLARDFADKPLFMTEY
GH30_NLL05159.1 230 NYAKYMDLSQ.........VYGIAHHLYNGGDANNPDSFNSNFESLARDFADKPLFMTEY
GH30_7_TcXyn30A 249 NILYEVQQAG.....GEHFFDVATWHNYQS.SPER......PFNVV.....GKPNIMTEW
GH30_7_TcXyn30B 252 AMIPGLQVVGPDGKSAEDYLSVVTGHGYSS.APTF......PLSTK......RRTWLTEW
GH30_7_TcXyn30C 244 TWTAQLASAG.....ATQYLARITSHWYAS.KGTS......PINTS......LRVWETEY
GH30_7_TrXynVI 245 TYTTNLVNAG.....STQYLSVITSHSYSS.DATS......PLSQTS.....LPKWNTEG
GH30_6_WP_028726386.1 276 GSKNYVRDILNLNTRITNYPIIAAGHGYVD.PVTKKDPAIEPFTKAES..KNVPVWLTEI
GH30_10_AcXbh30A 248 NSLNDPIAVKRV........DIVGAHNYGS.SYI.......PFTTTKS..KGKGIWMTEV
GH30_8_EcXyn30A 213 PVLKDSDASKYV........SIIGGHLYGT.TPKP.....YPLAQNA....GKQLWMTEH
GH30_8_CaXyn30A 220 PTLNDPTTAQYV........SIIGGHLYGS.PIRD.....YPLARNK....GKDIWMTEH
GH30_8_BsXyn30C 216 PILNDPQALANM........DILGTHLYGT.QVSQFP...YPLFKQKG..AGKDLWMTEV
GH30_8_CpXyn30A 220 PILNDATALDNM........DVLGCHFYGT.SVNNMA...YPLYQQKS..AGKELWMTEK
GH30_8_CpXyn30A
β6 α6 β7
α7 β8
GH30_3_AAO78418.1
GH30_3_AAO78418.1 341 S..........IGTWNSGRDLSKRLMEDMEEVALGTINNWC.....KGVIVWNLMLDND
GH30_1_AAA35880.1 381 CVGSKFWEQSVRLGSWDRGMQ............YSHSIITNLLY.HVVGWTDWNLALNPE
GH30_2_ABX45137.1 304 EC..........GTGDNSWE.........YAEYIFHLINHYFRNGATAYTYWNMILDDQ
GH30_9_LcGUS30 324 RC..........MMTDTPEE........AAHIMFGIIGNWLVHNGLSMITLWNLALDER
GH30_11_ShGbhA 297 CCSLGEAN.....GTGRGWSG.GYDPTITNALMFSGMVLQSFVLAGEPHYDFWTLVSNGI
GH30_5_BAC84995.1 313 GDSDASGK........................SMYQNLLLDFTWLHPTAWVYWQAIDGAG
GH30_4_PbFucA 331 SMLDAEPST...EAGFPASYD..AATYMDIALYMGKLIYSDIVYANNTSWSYWTAMAQEK
GH30_HHU50367.1 284 ..........................DQGTPFTTALLMHHSLVEEGVNAYFFWDLIWENS
GH30_WP_114299953.1 287 ..........................DQGTPFTTTQLIHNSLVEEGVSSYFFWDLIWDNS
GH30_NLM60409.1 281 ..........................DYGTPFTTAQLIYNSLVIEGVSAYFYWDLIWENS
GH30_AcXyn30B 281 ..........................DYGTPFTTAELIHNSLVVEGVSGYFYWDLIWENS
GH30_NLL05159.1 281 ..........................DYGTPFTTAELIHNSLVVEGVSGYFYWDLIWENQ
GH30_7_TcXyn30A 292 ADGSGPWNT..........TWDVSGQLAEGLQWALYMHNAFTNSDTSGYNHWWCAGGGA
GH30_7_TcXyn30B 299 TDLSGAFTP.........YTFFADGGAGEGMTWANHIQTAFVNANVSAFIYWIGAENST
GH30_7_TcXyn30C 286 ADLDDAFTT..........TWYSSGAANEGLTWANLIWQGVVEADLSAFLYWIGAQSNS
GH30_7_TrXynVI 288 .GPSTPFVK..........TWYSNGGTNEGFTWANKIAVAMVNAQLSAYLFWEGFEIQQ
GH30_6_WP_028726386.1 333 SDPHNS.................YSTSIEDGLNWAVKFHRYLCEANVSSIIWWAGALPDS
GH30_10_AcXbh30A 290 SDMNG..................NDTTINDGLRWAKEIHDFMTITEGNAWFYWWGACFKT
GH30_8_EcXyn30A 255 YVDSKQ...............SANNWTSAIEVGTELNASMVS.NYSAYVWWYIRRSY.
GH30_8_CaXyn30A 262 YLEGN...................DPGTCVKLAKEIHDCMTIGNMNAYVYWWISGDQ.
GH30_8_BsXyn30C 262 YYPNSDT...............NSADRWPEALDVSQHIHNAMVEGDFQAYVWWYIRRSY.
GH30_8_CpXyn30A 266 YFDDD...................TTGNIMNMSKEIHDSMVTGNMNAYIYWWITWPN.
GH30_8_CpXyn30A
α7 β8

```
GH30_3_AAO78418.1                                                          α8

GH30_3_AAO78418.1      385  RGPNREG........GCQTCYGAVDINNS...........DYKTIIRNSHYYIIAHLSS
GH30_1_AAA35880.1      428  GGPNWVR........NFVDSPIIVDITKD..............TFYKQPMFYHLGHFSK
GH30_2_ABX45137.1      344  DSTWGW............WQNSLFTITAD............KHEVRRNPEYYVMRHFSH
GH30_9_LcGUS30         365  GLPNAAD............STGREGVVTID.........HTTGKVQRNLEYFMLRNFGQ
GH30_11_ShGbhA         351  GCTPGDNSTCDPTVPNAEGWTDGVIYYDAN......YATNGNYELYLTKHFWTYKHFGN
GH30_5_BAC84995.1      349  W.....................GLIVGDND..........NLTLSSASTKYFVLAQLTR
GH30_4_PbFucA          386  WSQKNRF............YLLRLIDADDTDGGESYGDIRKGGTIEDSKSLWVLGNYSR
GH30_HHU50367.1        318  QR...................PFIQLENPW....DQSSWSSDKGYIITEFFHVFKHYSK
GH30_WP_114299953.1    321  QR...................PMVIVEPPF....NQNGWSNPQGYYKTDFYSSIQHYAK
GH30_NLM60409.1        315  QR...................PLVFIENPF....TPNSWTTQKGYILSDFYYTIQQYAK
GH30_AcXyn30B          315  QR...................PLVAIEVPR....NPEQWTTKEGYIISDFYYIMQQFAK
GH30_NLL05159.1        315  QR...................PLVFIEKPR....NPNEWTTEEGYIISDFYPIIQQYAK
GH30_7_TcXyn30A        341  DN.....................VLISITG............NSYEVSSRLWAFASYFR
GH30_7_TcXyn30B        349  TNS....................GMINLIN............DEVIPSKRFWSMASFSK
GH30_7_TcXyn30C        335  NAA....................GLVTLNG............STVQASGTLWAFAMFSR
GH30_7_TrXynVI         336  SQSG...................SHLIDAL..........DRQTATPSGIFWAFAMWSR
GH30_6_WP_028726386.1  376  GTNE...................GLIYIAK..........NRTDYETGKRYETFGNFTR
GH30_10_AcXbh30A       332  YNGE...................GLIQMDL..........NSKTYKVAKRLYTIGQFSR
GH30_8_EcXyn30A        296  .......................GLL...............TEDGKVSKRGYVMSQYAR
GH30_8_CaXyn30A        300  .......................NGLYN.............TRTNETYKKTYVMGQFSK
GH30_8_BsXyn30C        306  .......................GPM...............KEDGTISKRGYNMAHFSK
GH30_8_CpXyn30A        304  .......................GLA...............TSSGTIYKRAYVLGQFAK
GH30_8_CpXyn30A                                                            α8

                                     1    1

                              β4X          β5X        β6X        β7X
GH30_3_AAO78418.1

GH30_3_AAO78418.1      425  VVKPGAVRIATTGY....TDNGITCSAFENT.DGTYAFVLINNNEKSKKITVSDGQ....
GH30_1_AAA35880.1      465  FIPEGSQRVGLVAS....QKNDLDAVALMHP.DGSAVVVVLNRSSKDPVLTIKDPAV...
GH30_2_ABX45137.1      379  YVRPGAKVLGTTGH......FNSMAIAFRNP.DGTVVVVAQNALDEERPFEFADPDAAE.
GH30_9_LcGUS30         403  DVSVGATVIGSTNYTRDGYTGGLGSVAFLGT.AGDIAAHLYNPTAQPIQAAVTINGNGA.
GH30_11_ShGbhA         404  FVKPGSQRNIVTGDDA......SNTTLVVST.SDSYHVLAMNPATDPVNATLTFPDVVCA
GH30_5_BAC84995.1      377  HIRQGMQILTTPDV........NTAVAYDAG.SQKLVIVTANWG.SAQTITFDLTRARTA
GH30_4_PbFucA          433  FIRPGYQRINLEASNLG..INGLMGTAYLSPNQDQMVCVFVNMGYTRRNLSFDIDGYSI.
GH30_HHU50367.1        354  FTDPGYQRVAADCS.....VDDIKISAFVSPDHKSLTVVLINQGHNTADVALDLNGYAVN
GH30_WP_114299953.1    357  FTEPGYSRVKAESS.....GSNVSVTAFTSPGKDKLTLVLINKASSESTISLNLNGYTAD
GH30_NLM60409.1        351  FTDPGFKRVEANCN.....SNDVKVTAFVSPDESKLTMIFINTASAQNTVSLDLNGFSFN
GH30_AcXyn30B          351  FTDPGYKRVDASVS.....SNDIKVSAFVSPDKSKLTMIFINKGYSEHSVALDLKGYSSN
GH30_NLL05159.1        351  FTDPGYKRVKVSLN.....ASDVKASAFVSPDQSKLTMILINKASSENTVALDLNGYSSN
GH30_7_TcXyn30A        367  FARPGSVRIGATSS.....VENVYVSAYENK.NGTVSIPVINAAHFPYEVTIDLQGLKAR
GH30_7_TcXyn30B        376  FVRPNAQRVKATSS.....DASVTVSAFENT.NGVVAIQVINNGTSAASLTIDLGKTHKE
GH30_7_TcXyn30C        362  FIRPDAVRISTSGS.....PSNVNVGAFKNA.DGSIVVVAINNNG..NSETISLSGITA.
GH30_7_TrXynVI         366  YIRPGASRVATSGS.....LSNVIIGAFQNT.DRSIVVVFTNSGTSAQSARVSFSGFTP.
GH30_6_WP_028726386.1  406  YIPVGSRRISAEYN....TDQGYMVSGYKN..GNSFTAVAINTNDTEKTLDLLINNTQTT
GH30_10_AcXbh30A       362  FIRPGWQRIEATKN....PVSNVYVTAYKDPKTGKFAIVAINNGWSKQSITYTLKGFSPA
GH30_8_EcXyn30A        317  FVRPGALRIQATEN....PQSNVHLTAYKNT.DGKMVIVAVNTNDSDQMLSLNISNANVT
GH30_8_CaXyn30A        323  FIGNGYSRVDATNS....PQSNVYVSAYTG..NNKVVIVAINQGTYPVNQSFNVQNSTVS
GH30_8_BsXyn30C        327  FVRPGYVRIDATKN....PNANVYVSAYKG..DNKVVIVAINKSNTGVNQNFVLQNGSAS
GH30_8_CpXyn30A        325  FIRPGYKRVDATAT....PNTNVYVSAYTG..DNKAVIVAINTGTAAVSQKFNFQNGSAS
GH30_8_CpXyn30A
                              β2X          β3X        β4x        β5X

                                                      β8X        β9X
GH30_3_AAO78418.1

GH30_3_AAO78418.1      476  ..............................RHFAYDVPGKSVTSYRWAKSK.........
GH30_1_AAA35880.1      517  ..............................GFLETISPGYSIHTYLWHRQ..........
GH30_2_ABX45137.1      431  ..............................RGFKVTLAPRSFNTFVLD............
GH30_9_LcGUS30         461  ..............................NWQLVTVPPYGTVT..LHKSDAPLNTTNVP
GH30_11_ShGbhA         457  T....TAVRTSADE...DFATVDPATEATNGTWVLALAATSLTTFTFERGAC........
GH30_5_BAC84995.1      427  GSNGATVPRWSTQTGGGDQYRSYTDTKINNGKFSASFSSGQVQTFEVSGVVLQ.......
GH30_4_PbFucA          490  ....SHIDTYTTSE......TQNLEHASYAADETFNVAKGTVTTVVLSISPTAGIEHLT.
GH30_HHU50367.1        409  D....SAV.YRTVP...NGSERFALIGSLGAGHTLSMPAQSIVTVVVNSS..........
GH30_WP_114299953.1    412  T....SAV.YRTVF..SGTAERFAHLGSLQ.GNTVTMPAQSVVTVALGFP..........
GH30_NLM60409.1        406  K....SVI.YRTLE...NGNEKFAPAGSLT.GNTVTLPARSIVTVALGFD..........
GH30_AcXyn30B          406  K....SII.YRTLP...EGNERFKKVGSLF.GNTVTLPARSVVTVALGVD..........
GH30_NLL05159.1        406  K....SVV.YRTLS...NGTEGFKKVGSLS.GNTVTLPAQSVVTVAFGVS..........
GH30_7_TcXyn30A        421  ....KRVSTFLTDN..SHNVTLMDQSELHGSVLKATVPPRAVQVFWLE............
GH30_7_TcXyn30B        430  V...KKVVPWVTSN..DYDLEEMSEIDVKHNSFLASVPARSLTSFVTECE..........
GH30_7_TcXyn30C        413  ....SKVSAYYMDS..AVSSPSTFSATLNGGTVGGSLPARSMVTFVITTGSSGSA.....
GH30_7_TrXynVI         419  ....TSAAAYVTDN...SHTFSTTSAGLSGGAITVSVPSKGVVTVKLT............
GH30_6_WP_028726386.1  460  ....GPIVGHLTDA..THKWATLDTIQPVNNTYVVTLPAKSVVTFTGNVE..........
GH30_10_AcXbh30A       418  .....SVTPYTTSS..TQNLEKGSDITVNNSSFSFELAPNSITTFVGDTES.........
GH30_8_EcXyn30A        372  .....KFEKYSTSA.SLNVEYGGSSQVDSSGKATVWLNPLSVTTFVSK............
GH30_8_CaXyn30A        377  .....NVSSWVSSG.TLNMAKTNSNISAANGRFNASLPAQSVTTFVAD............
GH30_8_BsXyn30C        381  .....NVSRWITSS.SSNLQP.GTNLTVSGNHFWAHLPAQSVTTFVVNR...........
GH30_8_CpXyn30A        379  .....SVVSYVTDS.SRNMAA.GANIAVTNGSFTAQLPAQSITTFVGNTAPVVVEPIDAF
GH30_8_CpXyn30A
                              β6X   β7XA  β7XB    β8X        β9X
```

Fig. S1. Multiple sequence alignment of catalytic domain of *Ac*Xyn30B and the members from each GH30 subfamily. Four proteins most similar to *Ac*Xyn30B according to BlastP are also included and marked by a vertical bar on the left. Secondary structure elements of GH30_3 endo-β-1,6-glucanase BT3312 (from group 1) are shown on top (green) while secondary structure elements of GH30_8_*Cp*Xyn30A (from group 2) are depicted on bottom (blue). Catalytic acid/base and catalytic nucleophile (in *Ac*Xyn30B Glu171 and Glu279, respectively) are highlighted in magenta. The differences in the arrangement of the β_9-domain relative to the $(\beta/\alpha)_8$ barrel in the group 1 (subfamilies 1, 2, 3, 9) and the group 2 (subfamilies 4, 5, 6, 7, 8, 10, 11) are highlighted in yellow. While the first three β-strands of the β9-domain precede the $(\beta/\alpha)_8$ barrel in group 1 (top four sequences), just one β-strand of the β9-domain is located in front of the $(\beta/\alpha)_8$ barrel in group 2 (other sequences). The prokaryotic arginine, which is responsible for glucuronoxylan specificity of the typical GH30_8 members, and the corresponding residues in other sequences are green highlighted. A different arginine residue, which often plays a similar role in eukaryotic GH30_7 glucuronoxylanases, is highlighted in grey. The shorter β2-α2 regions in *Ac*Xyn30B and GH30_8 sequences are marked in cyan and the shorter α7 helix in *Ac*Xyn30B and related enzymes is depicted in orange. The blue region shows a longer β8-α8 segment in *Ac*Xyn30B and related enzymes.

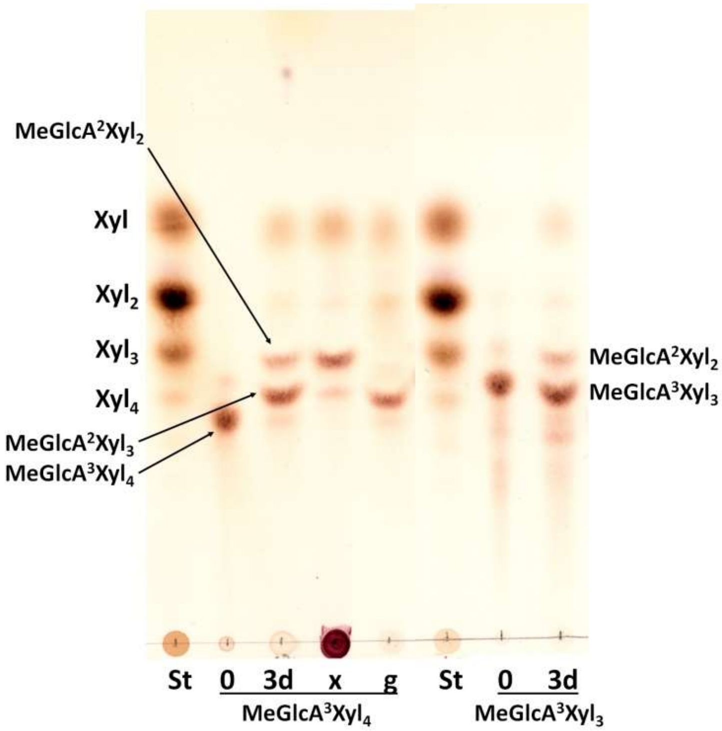

Fig. S2. TLC analysis of the hydrolysis products released from acidic XOs $MeGlcA^3Xyl_4$ and $MeGlcA^3Xyl_3$ by *Ac*Xyn30B after 3 days, and after subsequent addition of GH3 β-xylosidase (x) or GH67 α-glucuronidase (g). St – standards of linear XOs.

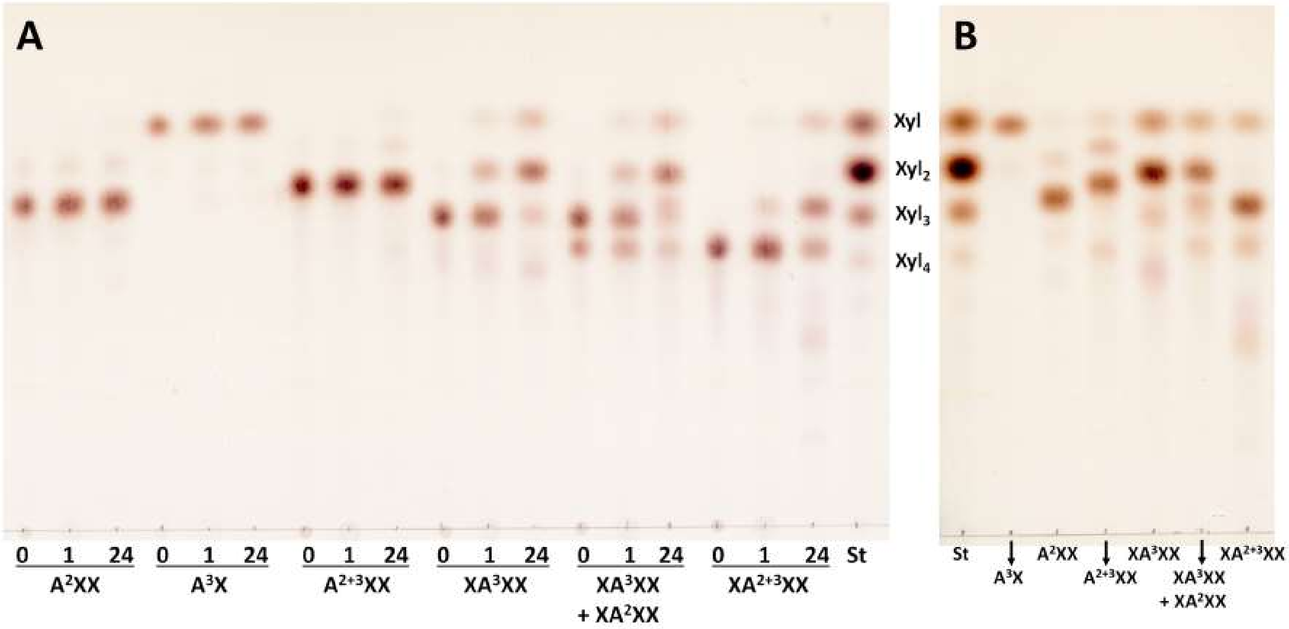

Fig. S3. TLC analysis of the products formed from arabinoxylooligosaccharides by *Ac*Xyn30B after 0 h, 1 h, and 24 h (**A**) and after 3 days (**B**). St – standards of linear XOs.

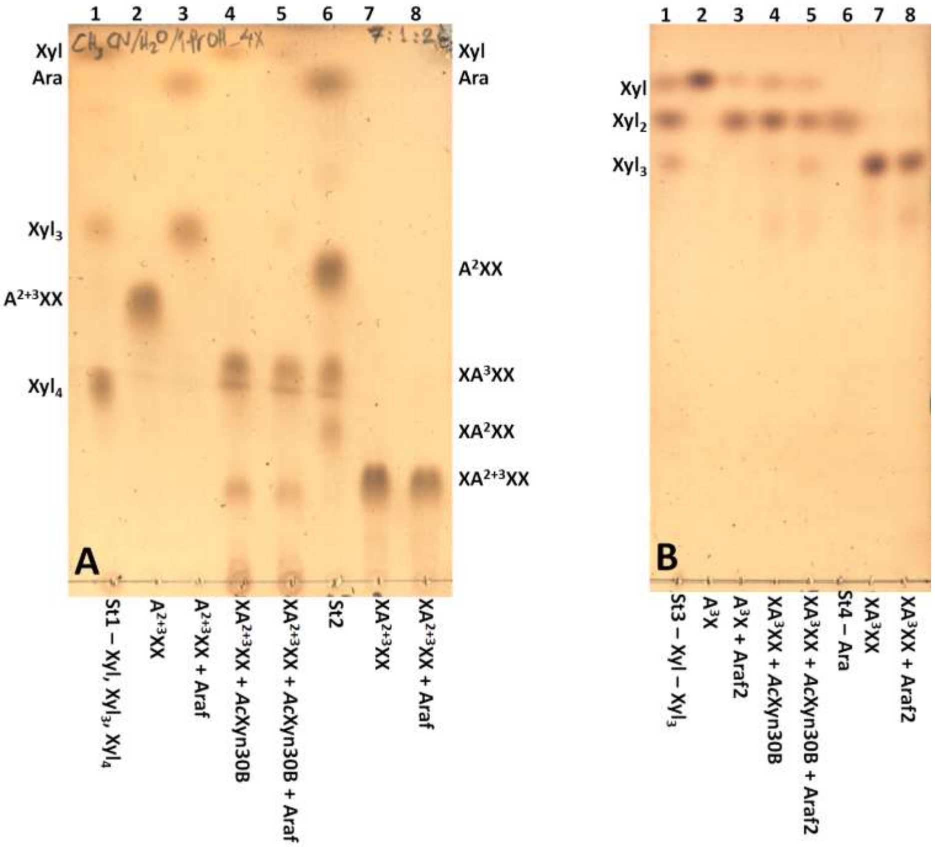

Fig. S4. Elucidation of the structure of products released by *Ac*Xyn30B from arabinoxylooligosaccharides (Ara-XOs) $XA^{2+3}XX$ (**A**) and $XA^{3}XX$ (**B**). In panel **A** GH51 α-arabinofuranosidase *Cj*Araf51A from *Cellvibrio japonicus* (Araf, ACE86344.1, Megazyme) was used. This α-arabinofuranosidase is able to release both Ara residues linked to the non-reducing end Xyl*p* residue of $A^{2+3}XX$ (lane 2), thus yielding Ara and Xyl_3 as final products (lane 3). In contrast, the enzyme does not attack $XA^{2+3}XX$ (lanes 7, 8) because the substitution is at the internal Xyl*p* residue. From $XA^{2+3}XX$ *Ac*Xyn30B forms xylose and an Ara-XO by one Xyl shorter than the substrate (lane 4). An addition of Araf to the hydrolysate didn't cause any change in the structure of Ara-XO formed by *Ac*Xyn30B (lane 5) which means that this Ara-XO doesn't have doubly arabinosylated Xyl*p* residue at the non-reducing end but is internally disubstituted. *Ac*Xyn30B thus release Xyl from the reducing end of $XA^{2+3}XX$ producing $XA^{2+3}X$. St – standards of linear XOs Xyl, Xyl_3 and Xyl_4 (lane 1), St2 – standards of Ara, $A^{2}XX$, $XA^{3}XX$, $XA^{2}XX$ (lane 6). The reactions were performed overnight at 35 °C using 0.1 % substrates and 0.01 mg/ml arabinofuranosidase (final concentration) in 50 mM sodium phosphate buffer, pH 6.0. The TLC plate was developed 4 times in the solvent system acetonitril/water/1-propanol 7:1:2 (v/v) and visualized using orcinol reagent (0.5% orcinol in 5% sulphuric acid in ethanol) and a heating at 105 °C.

In panel **B** a GH62 α-arabinofuranosidase from *Aspergillus nidulans* (Araf2, EAA59562.1, Megazyme) was used. This α-arabinofuranosidase rapidly converts $A^{3}X$ (lane 2) which is 3-arabinosylated at the non-reducing end terminal Xyl*p* residue, yielding Ara and Xyl_2 (lane 3). However, within the same period of time the enzyme does not attack $XA^{3}XX$, i.e. internally 3-arabinosylated XO (lanes 7, 8). From $XA^{3}XX$ *Ac*Xyn30B releases xylose and an Ara-XO by one Xyl shorter (lane 4) that is essentially resistant to the Araf2 (lane 5). Therefore, the Ara-XO generated from $XA^{3}XX$ by *Ac*Xyn30B carries internal 3-arabinosylated Xyl*p* residue. i.e

it has the structure XA^3X, and xylose released from the substrate XA^3XX originates from the reducing end. St3 – standards of linear XOs – Xyl, Xyl_2, Xyl_3 (lane 1), St4 – standard of Ara (lane 6). The reactions were performed for 1.5 h at 35 °C using 0.1 % substrates and 0.01 mg/ml arabinofuranosidase (final concentration) in 50 mM sodium phosphate buffer, pH 6.0. TLC plate was developed twice in 1-propanol/ethanol/water 7:1:2 (v/v) and visualized using orcinol reagent as mentioned above.
